# Histone methyltransferases EHMT1 and EHMT2 (GLP/G9A) maintain PARP inhibitor resistance in high-grade serous ovarian carcinoma

**DOI:** 10.1186/s13148-019-0758-2

**Published:** 2019-11-27

**Authors:** Zachary L. Watson, Tomomi M. Yamamoto, Alexandra McMellen, Hyunmin Kim, Connor J. Hughes, Lindsay J. Wheeler, Miriam D. Post, Kian Behbakht, Benjamin G. Bitler

**Affiliations:** 1 Division of Reproductive Sciences, Department of Obstetrics and Gynecology, University of Colorado School of Medicine, Aurora, CO 80045 USA; 2 Cancer Biology Graduate Program, University of Colorado, Aurora, CO 80045 USA; 3 Translational Bioinformatics and Cancer Systems Biology Laboratory, Division of Medical Oncology, Department of Medicine, University of Colorado Anschutz Medical Campus, Aurora, CO 80045 USA; 4 Medical Student Training Graduate Program, University of Colorado Anschutz Medical Campus, Aurora, CO 80045 USA; 5 Division of Gynecologic Oncology, Department of Obstetrics and Gynecology, University of Colorado School of Medicine, Aurora, CO 80045 USA; 6 Department of Pathology, University of Colorado School of Medicine, Aurora, CO 80045 USA

**Keywords:** HGSOC, Ovarian cancer, PARP inhibitor, Resistance, H3K9me2, EHMT1, EHMT2, DNA repair, Cell cycle

## Abstract

**Background:**

Euchromatic histone-lysine-*N*-methyltransferases 1 and 2 (EHMT1/2, aka GLP/G9A) catalyze dimethylation of histone H3 lysine 9 (H3K9me2) and have roles in epigenetic silencing of gene expression. EHMT1/2 also have direct roles in DNA repair and are implicated in chemoresistance in several cancers. Resistance to chemotherapy and PARP inhibitors (PARPi) is a major cause of mortality in high-grade serous ovarian carcinoma (HGSOC), but the contribution of the epigenetic landscape is unknown.

**Results:**

To identify epigenetic mechanisms of PARPi resistance in HGSOC, we utilized unbiased exploratory techniques, including RNA-Seq and mass spectrometry profiling of histone modifications. Compared to sensitive cells, PARPi-resistant HGSOC cells display a global increase of H3K9me2 accompanied by overexpression of EHMT1/2. EHMT1/2 overexpression was also observed in a PARPi-resistant in vivo patient-derived xenograft (PDX) model. Genetic or pharmacologic disruption of EHMT1/2 sensitizes HGSOC cells to PARPi. Cell death assays demonstrate that EHMT1/2 disruption does not increase PARPi-induced apoptosis. Functional DNA repair assays show that disruption of EHMT1/2 ablates homologous recombination (HR) and non-homologous end joining (NHEJ), while immunofluorescent staining of phosphorylated histone H2AX shows large increases in DNA damage. Propidium iodide staining and flow cytometry analysis of cell cycle show that PARPi treatment increases the proportion of PARPi-resistant cells in S and G2 phases, while cells treated with an EHMT1/2 inhibitor remain in G1. Co-treatment with PARPi and EHMT1/2 inhibitor produces an intermediate phenotype. Immunoblot of cell cycle regulators shows that combined EHMT1/2 and PARP inhibition reduces expression of specific cyclins and phosphorylation of mitotic markers. These data suggest DNA damage and altered cell cycle regulation as mechanisms of sensitization. RNA-Seq of PARPi-resistant cells treated with EHMT1/2 inhibitor showed significant gene expression changes enriched in pro-survival pathways that remain unexplored in the context of PARPi resistance, including PI3K, AKT, and mTOR.

**Conclusions:**

This study demonstrates that disrupting EHMT1/2 sensitizes HGSOC cells to PARPi, and suggests a potential mechanism through DNA damage and cell cycle dysregulation. RNA-Seq identifies several unexplored pathways that may alter PARPi resistance. Further study of EHMT1/2 and regulated genes will facilitate development of novel therapeutic strategies to successfully treat HGSOC.

## Background

High-grade serous ovarian carcinoma (HGSOC) is the most common epithelial ovarian cancer histotype and has one of the highest death-to-incidence ratios of all cancers [1]. High mortality is due primarily to frequent late stage diagnosis, high recurrence rates, and the development of therapy resistance. Over 80% of cases recur and ultimately present as chemoresistant disease.

Poly ADP ribose polymerase inhibitors (PARPi) were initially developed to treat homologous recombination (HR) DNA repair-deficient tumors (e.g., BRCA1/2-mutated). Mutation of BRCA1/2 often leads to a defective HR repair pathway and significantly increases the risk of developing HGSOC [2]. Analysis of HGSOC in The Cancer Genome Atlas (TCGA) predicts that close to 50% of HGSOC have some deficiencies in the HR pathway. The three approved PARPi (olaparib, rucaparib, and niraparib) were initially used for recurrent HGSOC with *BRCA1*/2 mutations as a third- or fourth-line therapy, but the SOLO1 clinical trial showed that first-line maintenance olaparib reduced risk of disease progression or death by 70% in newly diagnosed cases [3]. Further trials showed that BRCA-wildtype tumors also significantly benefit from PARPi [4, 5]. In the future, nearly all patients with HGSOC could receive olaparib. Unfortunately, acquired resistance is an emerging clinical problem that limits PARPi efficacy. Known mechanisms of PARPi resistance, such as *BRCA1*/*2* reversion mutations, restore HR but are found in only a small proportion of resistant cancers [6–9], suggesting that PARPi resistance has other causes that have yet to be explored [10].

Epigenetic regulation of transcriptional programming has been associated with chemo- and targeted-therapy resistance [11, 12]. Euchromatic histone-lysine *N*-methyltransferase 1 and 2 (EHMT1/2, also known as GLP and G9A, respectively) are epigenetic enzymes that promote transcriptional repression through histone modifications [13–15]. Functionally, EHMT1 and EHMT2 heterodimerize and interact with multi-zinc finger protein ZNF644 to form a complex which catalyzes s-adenosylmethionine (SAM) molecules to H3K9 to form mono- and di-methylated K9 (H3K9me1/H3K9me2). EHMT1/2 both contain acidic regions, ankryin protein-protein interaction domains, and share 80% homology in their methyltransferase SET-domains [13, 16, 17]. Beyond canonical roles in transcriptional repression, the EHMT1/2 complex also directly promotes DNA damage repair via recruitment of BRCA1, 53BP1, and other factors involved in HR and non-homologous end joining (NHEJ) [18, 19]. In the context of ovarian cancer, EHMT2 is frequently amplified and overexpressed [20, 21] and high expression correlates with aggressive peritoneal metastasis and poorer overall survival [22]. Prior to this study, the roles of EHMT1/2 in chemotherapy and PARPi resistance have not been examined in HGSOC.

In this report, we employed an unbiased proteomic approach to profile the epigenetic landscape of PARPi-resistant HGSOC cells. We found an enrichment of H3K9me2 compared to matched PARPi-sensitive cells. Analysis of a tissue microarray (TMA) shows that high H3K9me2 is associated with poorer overall survival. Parallel transcriptome analysis of H3K9-associated epigenetic enzymes revealed a significant increase in EHMT1/2 expression. Tumors from a patient-derived xenograft (PDX) model of PARPi-treated HGSOC also exhibited upregulation of EHMT1/2. Using both genetic and pharmacologic approaches to disrupt EHMT1/2 in PARPi-resistant cells, we resensitized cells to PARPi. Mechanistically, EHMT1/2 disruption did not increase cell death or apoptosis, but did promote increased DNA damage, ablated both HR- and NHEJ-mediated DNA damage repair, and altered cell cycle and transcriptional regulation.

## Results

### H3K9me2 is globally increased in PARPi-resistant HGSOC cells and correlates with poorer overall survival

**Fig. 1:**
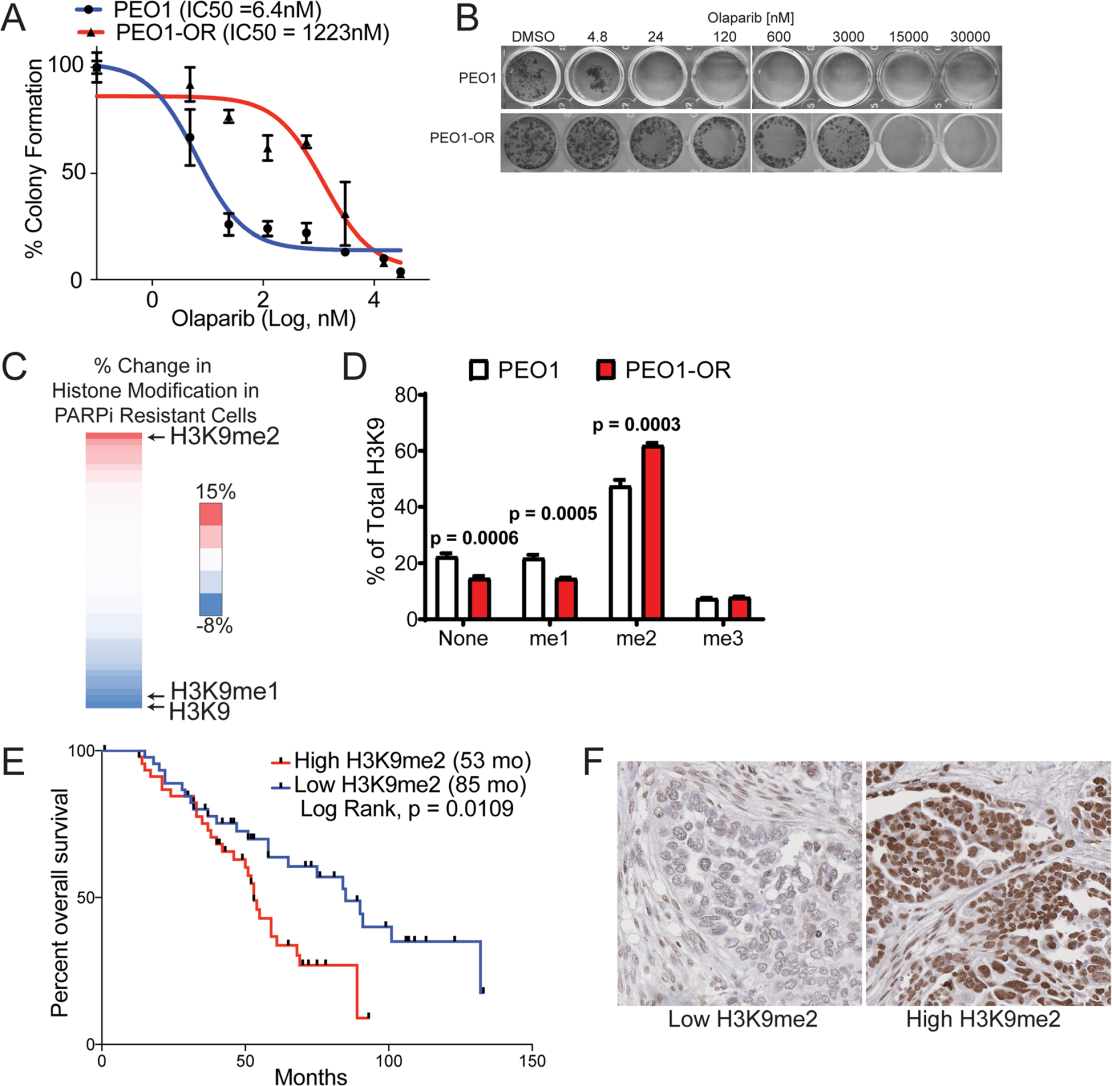
Olaparib-resistant HGSOC cells have increased H3K9me2. **a** PEO1 (TP53 and BRCA2-mutated) were treated in a step-wise fashion with increasing doses of olaparib. PEO1 sensitive and resistant (PEO1-OR) cells were plated in a 24-well plate and treated with increasing doses of olaparib for 12 days. Cells were stained with crystal violet. **b** Olaparib resistance was confirmed with a dose response colony formation assay. Dose response curves of PEO1 and PEO1-OR are graphed with IC50 indicated. **c** Histone modifications of PEO1 and PEO1-OR cells were analyzed by mass spectrometry. Heat map shows percent change of each modification in PEO1-OR relative to PEO1. Arrows indicate he most downregulated (unmodified H3K9, H3K9me1) and upregulated (H3K9me2) modifications. **d** Profile of H3K9 methylation in PEO1 and PEO1-OR cells (mean percent of total H3K9 ± SD, *n* = 3, unpaired *t* test). **e** Kaplan-Meier analysis of H3K9me2 staining in a TMA versus overall patient survival. **f** Representative images of Low and High H3K9me2 staining in the TMA

To examine the epigenetic landscape of PARPi-resistant cells, we subjected a PARPi-sensitive HGSOC cell line (PEO1, *TP53/BRCA2*-mutated) to step-wise dose escalation of olaparib to select for resistant cells. Olaparib resistance was confirmed in PEO1-olaparib-resistant (PEO1-OR) cells using a dose response colony formation assay. PEO1-OR cells were 191X more resistant to olaparib compared to parental cells (Fig. 1a, b). Notably, these cells lack previously known mechanisms of PARPi-resistance, including *BRCA2* reversion or *53BP1* loss or mutation [23]. Histones from PEO1 and PEO1-OR were isolated and 44 different histone H3 and H4 modifications were examined via mass spectrometry. H3K9me2 was significantly enriched in PEO1-OR cells compared to PEO1 cells (Fig. 1c, d). Conversely, H3K9 and H3K9me1 were significantly depleted in PEO1-OR cells compared to PEO1 (Fig. 1c, d). H3K9me3 was not significantly changed in PEO1-OR suggesting an increase in methyltransferase activity rather than demethylation activity. A full spreadsheet of mass spectrometry results is available in Additional file 1. We confirmed the mass spectrometry approach through immunoblot for the histone modifications showing the largest changes between PEO1 and PEO1-OR. We performed immunoblots using histone extracts from PEO1 and PEO1-OR, then performed densitometry analysis and normalized to total H3. In agreement with mass spectrometry, H3K9me1 was decreased in PEO1-OR by 8%, H3K9me2 was increased by 20%, and H3K14ac was increased by 11%. H3K27me3, which was relatively unchanged in our mass spec data, was also unchanged in Western blot (Additional file 2: Figure S1).

To correlate the in vitro H3K9me2 findings to clinically relevant specimens, we performed immunohistochemical staining for H3K9me2 using a TMA of serous tumors (tumor and patient details in Additional file 3). Slides were blinded and H3K9me2 staining was scored from 0 to 3, including half units. Scores < 2 were considered “Low” while scores ≥ 2 were considered “High.” We generated a Kaplan-Meier (K-M) survival curve by correlating scores to overall patient survival, and we observed that high H3K9me2 staining correlated with poorer overall survival (Fig. 1e). Examples of low and high staining are shown in Fig. 1f. H3K9me2 staining within stromal regions was consistent across samples, indicating that changes in H3K9me2 staining intensity were specific to tumor regions (Additional file 2: Figure S2). Although the TMA contains additional samples, to avoid confounding factors in our analysis, we used only the 92 primary, chemonaïve tumors for the K-M curve.

### EHMT1 and EHMT2 are overexpressed in PARPi-resistant HGSOC cell lines and patient-derived ascites

**Fig. 2:**
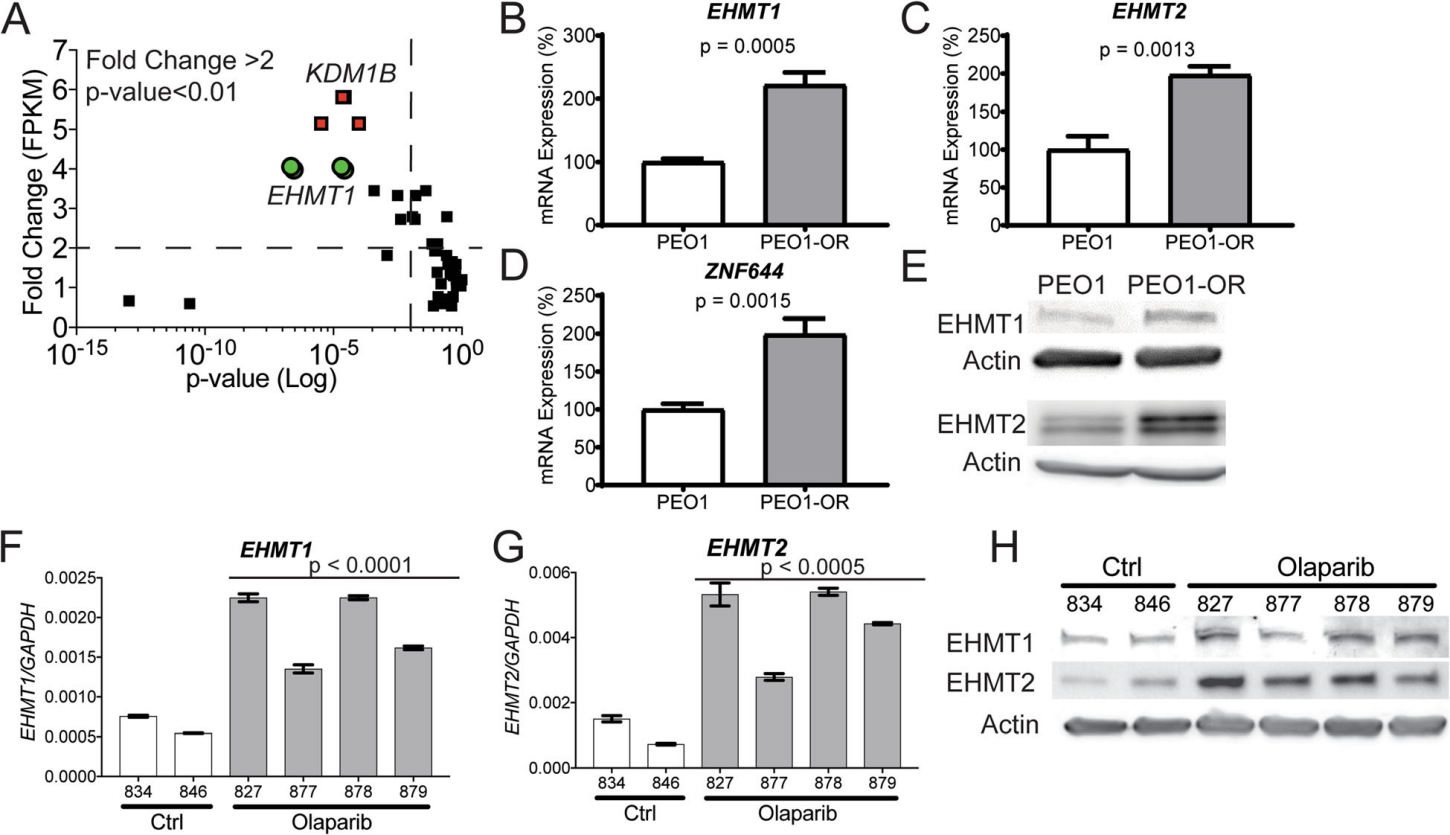
Histone methyltransferases EHMT1 and EHMT2 are upregulated in olaparib-resistant HGSOC. **a** Four olaparib resistant clones of PEO1-OR were analyzed by RNA-Seq. Of all enzymes involved in H3K9 methylation, *EHMT1* (red squares), and *KDM1B* (green circles) were significantly changed in all four populations of PEO1-OR relative to PEO1. **b**–**d** RT-qPCR analysis of histone methyltransferases *EHMT1* and *EHMT2*, and zinc-finger gene *ZNF644* in PEO1 and PEO1-OR cells (mean ± SD, *n* = 3, unpaired *t* test). **e** Protein lysates from PEO1 and PEO1-OR cells were analyzed by immunoblot for EHMT1, EHMT2, and Actin loading control. **f**, **g** Patient-derived HGSOC ascites cells were injected intraperitoneally into NSG mice. After 21-day treatment with olaparib or vehicle control, mice were sacrificed and ascites cells were collected and analyzed by RT-qPCR for *EHMT1* and *EHMT2* (mRNA expression is normalized to *GAPDH* and plotted as mean ± SD, *n* = 3 technical PCR replicates, unpaired test; numbers below bars are mouse ear-tag numbers). **h** Protein lysates from ascites cells were analyzed by immunoblot for EHMT1, EHMT2, and Actin loading control (ear tag numbers correspond with mRNA data)

We performed transcriptomic analysis with RNA-Seq of four clonal populations of PEO1-OR cells compared to PEO1 cells. We examined 13 known epigenetic regulators of H3K9 methylation. We observed that *EHMT1* was significantly upregulated in all four of the PEO1-OR clonal populations (Fig. 2a). Utilizing RT-qPCR, we confirmed that *EHMT1* was significantly upregulated (Fig. 2b). EHMT1 functions in a complex so we investigated the mRNA expression of complex subunits *EHMT2* and *ZNF644*, both of which were found to be significantly upregulated in PEO1-OR cells (Fig. 2c, d). Consistent with mRNA expression, both EHMT1 and EHMT2 protein expression were elevated in PEO1-OR cells compared to PEO1 (Fig. 2e). Short-term exposure of PARPi-sensitive PEO1 parental cells to olaparib does not induce elevated EHMT1/2 expression (Additional file 2: Figure S3) indicating that the observed increases in PEO1-OR are due to selection for high EHMT1/2 expressing cells that have a survival advantage in olaparib. Lysine demethylase *KDM1B* was also upregulated in the four PEO1-OR populations (Fig. 2a), but has yet to be validated in follow-up experiments.

Recurrent, PARPi-insensitive HGSOC is difficult to evaluate because in current clinical practice, ascites, and tumors from such patients are rarely collected. Therefore, we utilized a patient-derived xenograft (PDX) model of HGSOC to establish an olaparib-insensitive model. Following intraperitoneal injection of primary HGSOC ascites samples into immunocompromised NOD SCID gamma (NSG) mice, tumor-bearing mice were treated daily with olaparib or vehicle control for 21 days and the mice were monitored for 2 months (design schematic in Additional file 2: Figure S4). Olaparib-treated cells were subsequently shown to be highly olaparib-resistant [23]. Ascites cells were isolated from the control and olaparib-treated mice and examined for EHMT1/2 mRNA and protein expression. EHMT1/2 mRNA and protein expression were significantly upregulated in the olaparib-treated ascites cells compared to vehicle control (Fig. 2f–h).

### EHMT2 correlates with HGSOC progression and chemoresistance, and combined EHMT1/2 correlates with poor patient outcomes

**Fig. 3:**
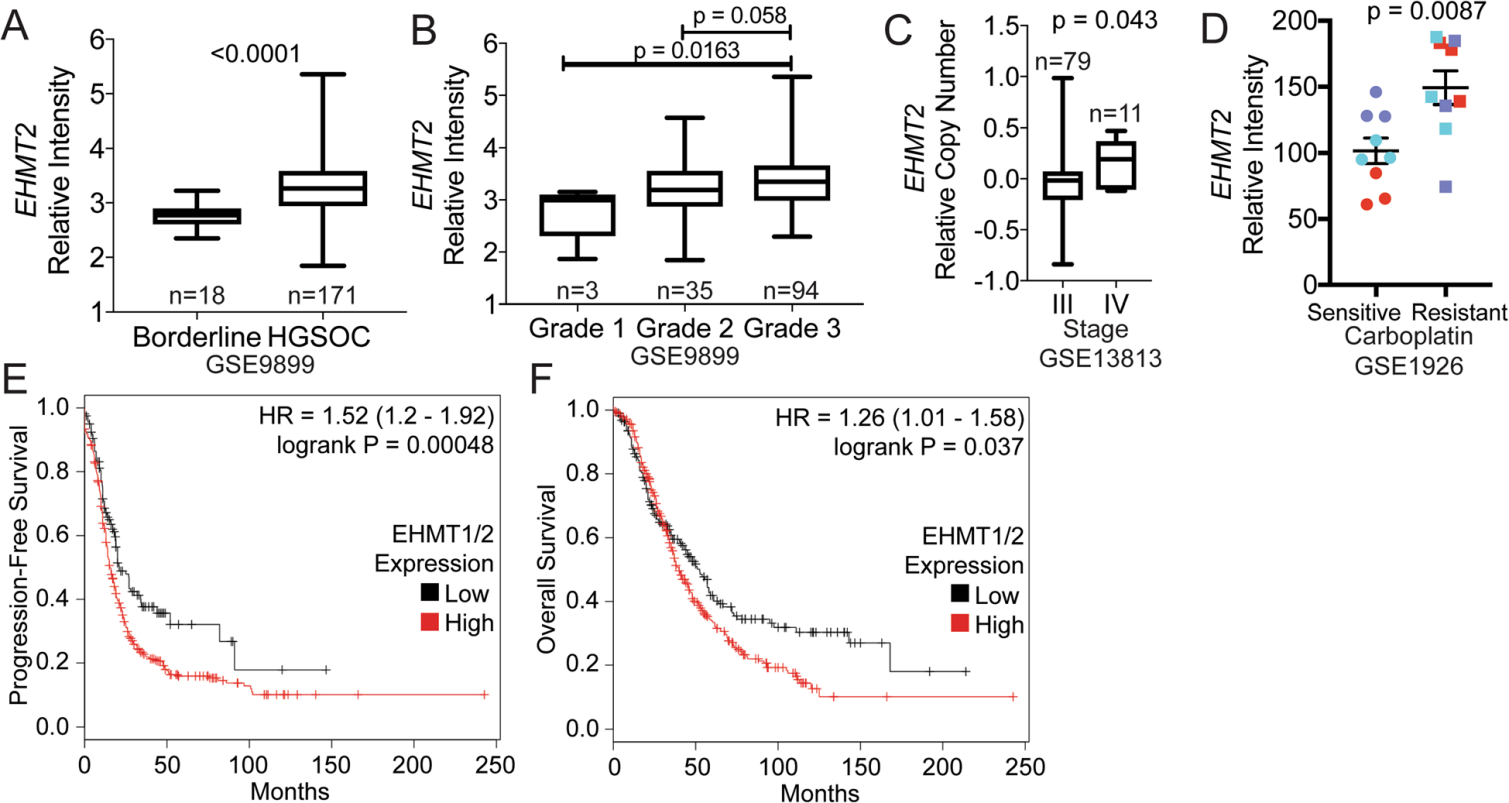
*EHMT2* is upregulated in advanced and chemoresistant HGSOC, and elevated *EHMT1*/*2* correlates with poorer clinical outcomes. **a** EHMT2 mRNA expression in Borderline versus HGSOC tumors. **b** EHMT2 mRNA expression in grade 1, 2, and 3 ovarian tumors. **c** Relative EHMT2 copy number correlated to ovarian cancer stage III or IV tumors. **d** Relative intensity of EHMT2 in carboplatin sensitive (*n* = 3) and resistant (*n* = 3) HGSOC tumors. Tumors analyzed in triplicate are color-coded. **e**, **f** Analysis of TCGA microarray data. A combined median expression was calculated for *EHMT1* and *EHMT2* to distinguish patients with low or high *EHMT1*/*2* expression. Kaplan-Meier curves were generated against (**e**) progression-free survival (N = 614) and (**f**) overall survival (*N* = 655)

We examined EHMT2 in publicly available datasets. EHMT2 is significantly upregulated in HGSOC relative to borderline tissue (Fig. 3a), in higher grade and stage of HGSOC (Fig. 3b, c), and in carboplatin-resistant tumors (Fig. 3d). While numbers are relatively small, these datasets are consistent with previous reports [20–22] and indicate a trend of elevated EHMT2 associated with advanced ovarian cancer and also suggest a link to therapy resistance. Complex subunits EHMT1 and ZNF644 were also significantly upregulated in HGSOC relative to borderline tissue, but were unaffected by grade, stage, or carboplatin resistance (Additional file 2: Figure S5). We also analyzed a TCGA microarray dataset of ovarian tumors and analyzed survival using K-M Plot [24]. We calculated a median combined *EHMT1*/*2* expression to differentiate high- and low-expressing tumors, then generated K-M plots compared to progression-free survival (PFS, *N* = 614) and overall survival (OS, *N* = 655). For both PFS and OS, higher *EHMT1*/*2* expression correlates with worse outcomes (Fig. 3e, f).

### Knockdown or inhibition of EHMT1/2 restores PARPi sensitivity

**Fig. 4.**
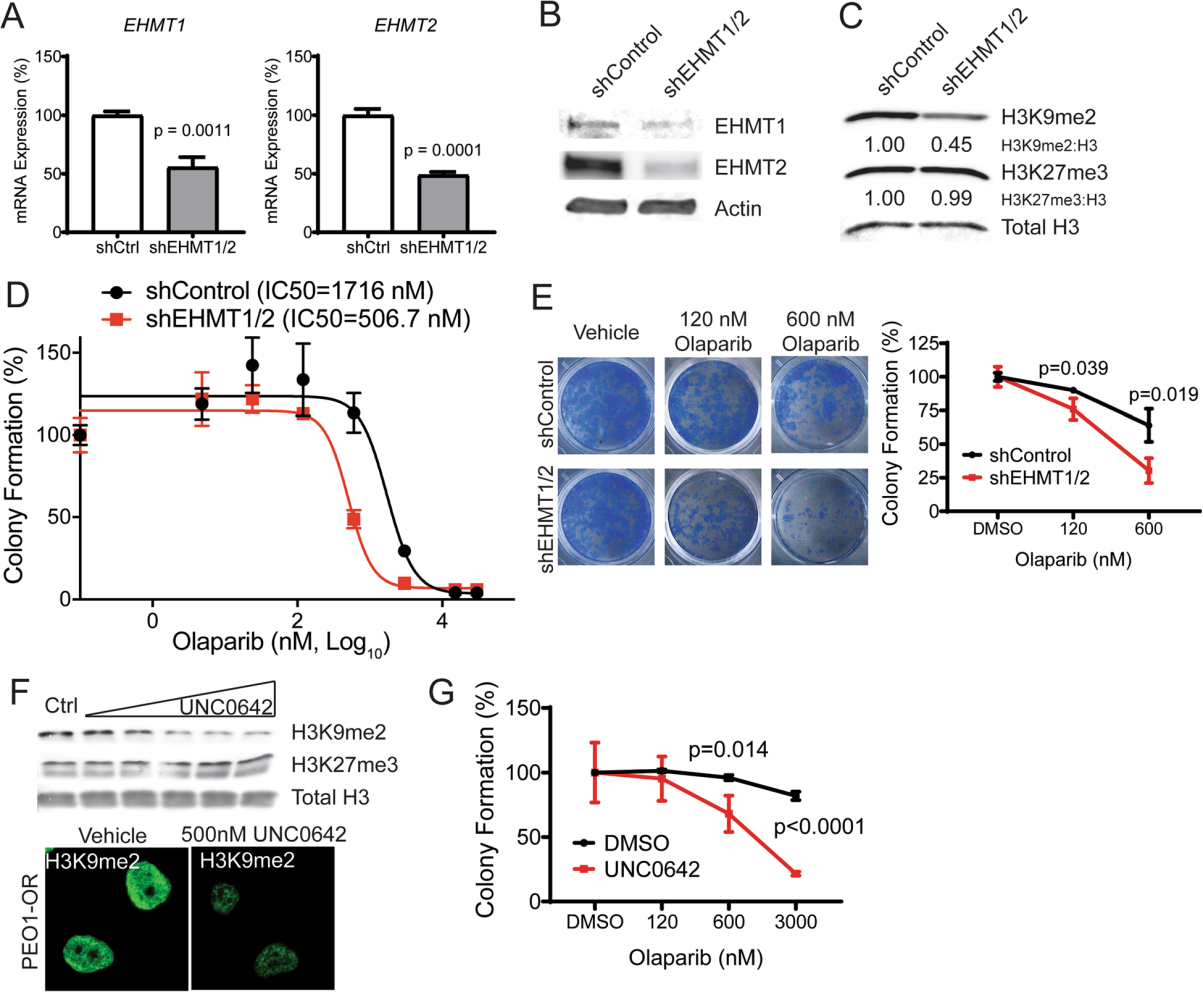
Knockdown or inhibition of EHMT1/2 resensitizes PEO1-OR cells to olaparib. **a** PEO1-OR cells were transduced with lentivirus carrying shRNAs targeting both EHMT1 and EHMT2 or Scramble control. Knockdown of *EHMT1* and *EHMT2* mRNA was analyzed by RT-qPCR (mean ± SD, *n* = 3, unpaired *t* test). **b** Knockdown of EHMT1 and EHMT2 protein was analyzed by immunoblot. Actin was used as loading control. **c** Histone extracts of double knockdown cells were analyzed by immunoblot for H3K9me2 and compared to Scramble control cells. Total H3 was used as loading control and for densitometry analysis. **d** Resensitization of EHMT1/2 double knockdown PEO1-OR cells was analyzed by dose response colony formation assays and compared to Scramble control cells. Dose response curves are graphed with IC50 indicated. **e** Cells from dose response assay were stained with crystal violet. Colony formation is plotted (mean ± SD of three wells, unpaired *t* test). **f** PEO1-OR cells were treated for 72 h with DMSO control or EHMT1/2-specific methyltransferase inhibitor UNC0642 at two-fold dilutions from 2 μM to 125 nM. H3K9me2 was analyzed by immunoblot or immunofluorescence. To confirm drug specificity, H3K27me3 was analyzed my immunoblot. Total H3 was used as loading control. IF images are representative from DMSO control and 500 nM treatment. **g** Resensitization of PEO1-OR cells co-treated with 1 μM UNC0642 was analyzed by dose response colony formation assays and compared to DMSO control treatment. Colony formation is plotted (mean ± SD of four wells, unpaired *t* test)

To determine if PARPi resistance is dependent on increased EHMT1/2 protein expression and activity, we disrupted EHMT1/2 using genetic and pharmacologic approaches and evaluated olaparib sensitivity. We first performed single knockdowns of EHMT1 or EHMT2 (Additional file 2: Figure S6). PEO1-OR cells were transduced with lentivirus encoding a non-targeting scrambled shControl, or one of two independent shRNAs targeting EHMT1 or EHMT2. While EHMT1 or EHMT2 mRNA and protein were individually reduced by all shRNAs (Additional file 2: Figure S6A-B), only EHMT2 sh#1 effectively reduced H3K9me2 (Additional file 2: Figure S6C). Olaparib dose response assays showed that knocking down the individual EHMT1 and EHMT2 subunits resensitized PEO1-OR cells to olaparib by a maximum of 1.3× and 2.4×, respectively, compared to PEO1-OR shControl (Additional file 2: Figure S6D-E). We surmised that single knockdown of EHMT1 or EHMT2 may not be an effective strategy due to overlapping histone methyltransferase functions [15], and that each subunit may compensate for loss of the other. We therefore chose to knock down both subunits within the same cells. We transduced PEO1-OR cells with lentivirus encoding the control shRNA, or a combination of both shEHMT1#1 and shEHMT2#1. To ensure double knockdown, we used a very high virus titer and multiplicity of infection. RT-qPCR and immunoblot confirmed EHMT1 and EHMT2 knockdown (Fig. 4a, b). Unlike single knockdowns, Western blot confirmed that knocking down both EHMT1/2 subunits in PEO1-OR resulted in a depletion of H3K9me2, while unrelated H3K27me3 was unaffected (Fig. 4c). We then evaluated olaparib response following the simultaneous knock down of EHMT1/2. We observed that the EHMT1/2-double knockdown cells were 3.4× more sensitive to olaparib compared to the PEO1-OR shControl (Fig. 4d), a marked improvement over single knockdowns. The differential response to olaparib in PEO1-OR shEHMT1/2 cells was clearly observed at 120 and 600 nM olaparib (Fig. 4e).

We next determined if pharmacologic inhibition of EHMT1/2 methyltransferase activity recapitulates results from knockdown experiments and restores olaparib sensitivity. We pre-treated PEO1-OR cells with highly-specific EHMT1/2 inhibitor UNC0642 [25] for 48 h and evaluated H3K9me2 abundance via immunoblot and immunofluorescence. UNC0642 treatment reduced H3K9me2 in a dose-dependent fashion, but unrelated H3K27me3 was unaffected (Fig. 4f). Dose-response colony formation assays show that co-treatment with UNC0642 resensitized PEO1-OR cells to olaparib (Fig. 4g), compared to cells treated only with olaparib. Differential response was particularly notable at 600 and 3000 nM olaparib. To determine if EHMT1/2 also convey a broad drug-resistant phenotype to DNA damaging agents, we performed combined UNC0642/cisplatin dose response assays. PEO1-OR cells were relatively sensitive to cisplatin, which was not affected by UNC0642 treatment, suggesting a specific resistance to olaparib in these cells (Additional file 2: Figure S7A). We also performed combined UNC0642/olaparib dose response assays in PARPi-sensitive PEO1 parental HGSOC cells. We noted no difference in olaparib sensitivity between olaparib alone or in combination with 1 μM UNC0642 (Additional file 2: Figure S7B), suggesting that PEO1-OR cells may have developed a dependence on EHMT1/2 that is not present in the parental, sensitive line. To determine if EHMT1/2 inhibition could sensitize BRCA1/2-wildtype cells, we performed combination UNC0642/olaparib dose response assays in PARPi-resistant OVCA433-OR cells [23] which are *TP53*-mutant, *BRCA1*/*2*-wildtype. Despite a lack of elevated EHMT1/2, these cells show a marked elevation of H3K9me2 relative to parental OVCA433 cells (Additional file 2: Figure S7C-D). One micromolar UNC0642 in combination with olaparib reduced resistance by 1.9× compared to olaparib alone (Additional file 2: Figure S7E), suggesting that EHMT1/2 inhibition may be effective in some *BRCA1*/*2*-wildtype tumors, particularly those with elevated H3K9me2, but is likely most effective in the context of HR-deficient tumors.

### Inhibition of EHMT1/2 alters cell cycle regulation but does not induce apoptosis or senescence

**Fig. 5:**
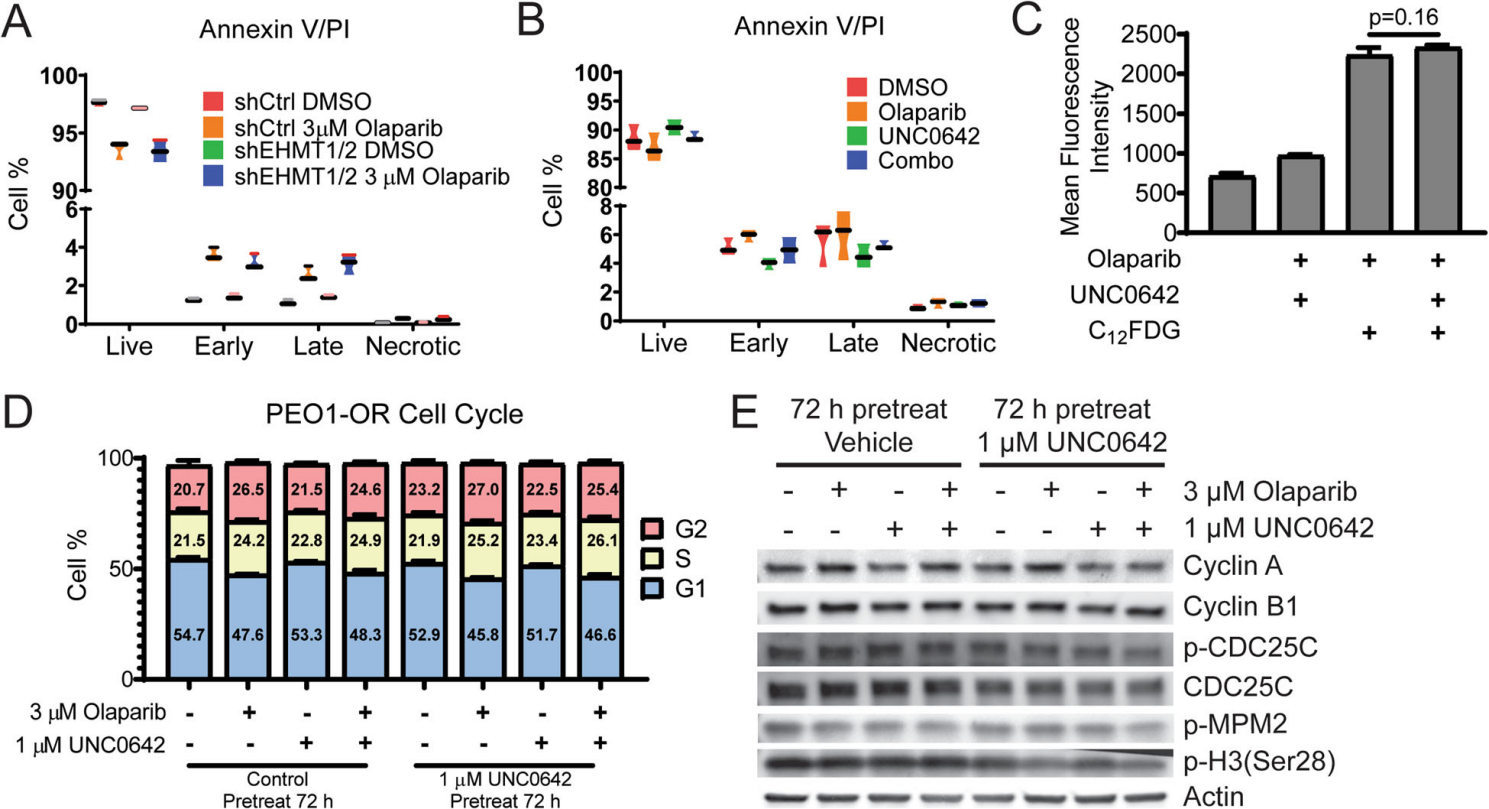
Disruption of EHMT1/2 in PEO1-OR cells alters cell cycle regulation, but not apoptosis or senescence. **a** Scramble shRNA and EHMT1/2 double knockdown PEO1-OR cells were treated with 3 μM olaparib for 72 h, then stained with Annexin V/PI followed by flow cytometry to assay for apoptosis and necrosis. **b** PEO1-OR cells were treated with 3 μM olaparib, 1 μM UNC0642, or a combination, and then assayed as in (**a**). For AnnexinV/PI analyses, cell % are plotted as violin plots with graphed means, *n* = 3. **c** β-galactosidase activity was assessed by detection of fluorescent C_12_FDG cleavage product in PEO1-OR cells treated as shown. Cells were analyzed using flow cytometry (mean ± SD, 50,000 cells per test, *n* = 3, unpaired *t* test). **d** PEO1-OR cells were treated as shown, then fixed and stained with propidium iodide for DNA content and analyzed by flow cytometry. Percentages of cells in G1, S, and G2 were calculated by the Dean-Jett-Fox method in FlowJo 10 (mean ± SD, 50,000 cells per test, *n* = 3). **e** Immunoblot analysis for cell cycle proteins using total protein lysates from PEO1-OR cells treated as shown

Our dose response colony formation assays showed that EHMT1/2 disruption lead to sensitization of PEO1-OR cells to olaparib. We next sought to determine if sensitization was due to cytotoxic (e.g., apoptosis) and/or cytostatic (e.g., senescence or reduced proliferation) effects of EHMT1/2 knockdown or UNC0642 treatment. We first used Annexin V/PI assays to measure apoptotic responses in PEO1-OR shControl versus shEHMT1/2 olaparib-resistant cells. As expected, treating shControl cells with a relatively high dose of olaparib (3 μM) for 72 h resulted in only ~ 5% total apoptotic cells. Olaparib treatment of shEHMT1/2 cells had a similar apoptotic response compared to shControl (Fig. 5a). Consistent with the EHMT1/2 knockdown, combining olaparib with 1 μM UNC0642 treatment did not significantly induce apoptosis (Fig. 5b).

We next assayed senescence using an assay of beta-galactosidase (β-gal) activity. Senescent cells have an increase in β-gal activity [26] that we measured using the substrate C_12_FDG. Cleavage of C_12_FDG by β-gal generates a fluorescent product that can be detected through flow cytometry. To determine if EHMT1/2 inhibition induced senescence, PEO1-OR cells were treated with UNC0642 and/or olaparib for 72 h and then incubated with C_12_FDG. β-gal activity was not significantly different following the inhibition of EHMT1/2 in combination with olaparib suggesting that senescence was not induced (Fig. 5c). To determine if EHMT1/2 inhibition altered cell cycle regulation, we pre-treated PEO1-OR cells for 72 h with vehicle control or UNC0642, then treated with olaparib, UNC0642, or combination for an additional 72 h. We then fixed cells and stained with propidium iodide (PI) for DNA content. Flow cytometry analysis allowed for examination of G1, S, and G2 phases (Fig. 5d). We observed that olaparib treatment alone significantly decreased G1%, while increasing S% and G2%. These changes were not observed with UNC0642 alone. Relative to olaparib alone, combined treatment with UNC0642 slightly decreased G2% and slightly increased G1% and S%, suggesting that a proportion of combination-treated cells are halting in G1 or are not completing DNA synthesis.

To further analyze cell cycle, we used the same conditions as for PI staining and then examined protein expression of several cell cycle regulators by immunoblot (Fig. 5e), including Cyclin A (present in S and G2, degraded in M), Cyclin B1 (G2/M-specific), phospho-CDC25C and total CDC25C (a tyrosine phosphatase that directs de-phosphorylation of Cyclin B-bound CDC2 and triggers entry into mitosis), and mitotic markers phospho-MPM2 (a protein motif that is phosphorylated in over 50 proteins) and phospho-H3(Ser28). We observed that olaparib alone increased Cyclin A and Cyclin B1, which is consistent with our observed increases in G2%. Conversely, UNC0642 alone reduced Cyclin A and Cyclin B1. When combined with olaparib, UNC0642 partially prevented the observed increases in Cyclin A and Cyclin B1 due to olaparib. Pretreatment with UNC0642 reduced total and phosphorylated CDC25C, and combined treatment with olaparib/UNC0642 showed the greatest reduction in both, indicating reduced entry into mitosis. Consistent with these data, combined treatment also reduced levels of p-MPM2 and p-H3(Ser28). Densitometry analyses of immunoblots are shown in Additional file 2: Figure S8. Taken together, these data suggest that combined PARP and EHMT1/2 inhibition may sensitize PARPi-resistant cells in a cytostatic manner by preventing entry into mitosis and reducing proliferation.

### Disruption of EHMT1/2 induces DNA damage, ablates DNA repair, and causes large transcriptional changes in survival pathways

**Fig. 6:**
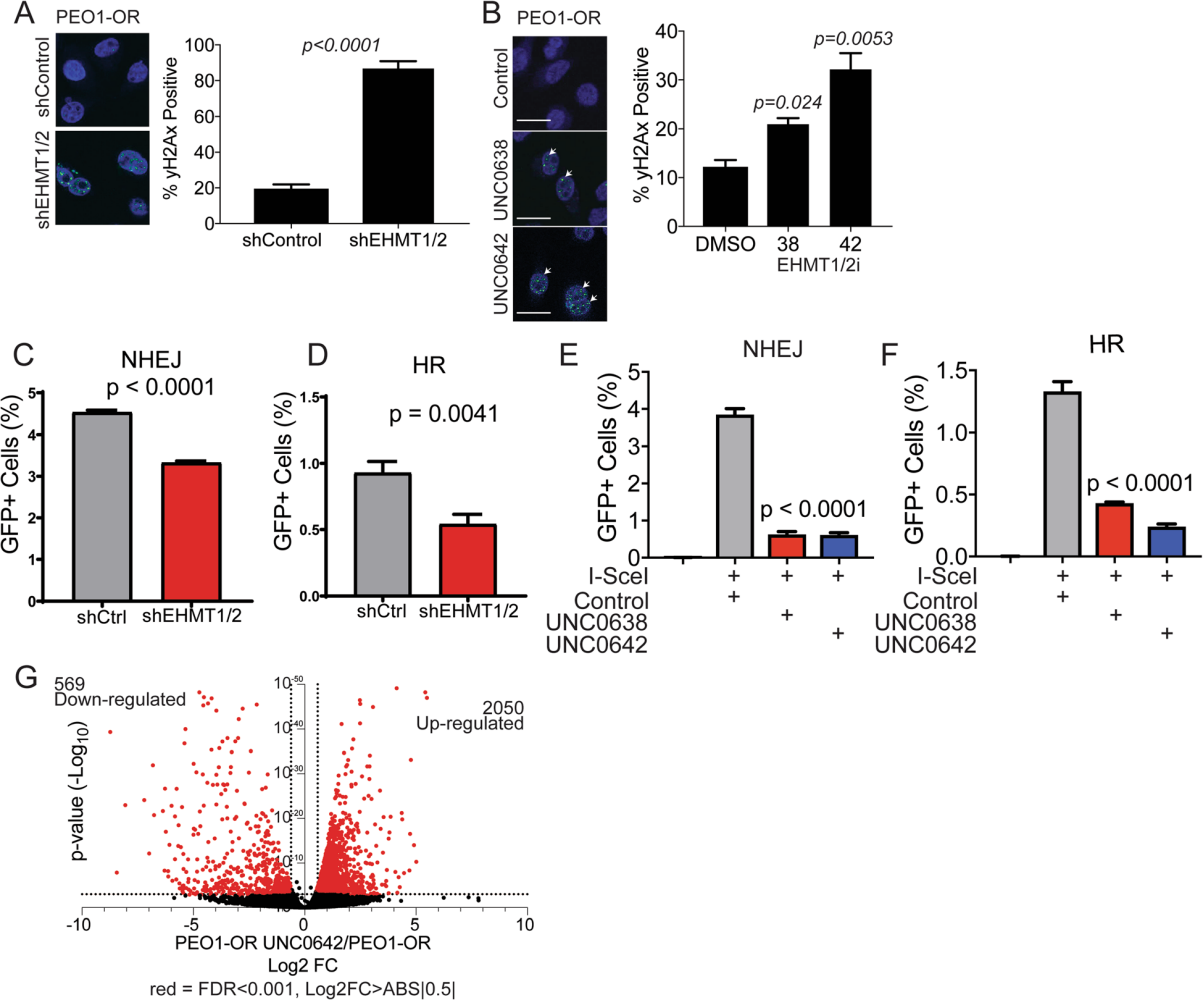
Disruption of EHMT1/2 in PEO1-OR cells impairs DNA repair and significantly alters gene expression. **a** Immunofluorescence staining for DNA damage marker γH2AX was performed on PEO1-OR shControl or shEHMT1/2 double knockdown cells. Nuclei were counterstained with DAPI. **b** Staining for γH2AX as in (**a**), except cells are PEO1-OR treated for 72 h with 1 μM UNC0638, UNC0642, or DMSO control. For (**a**, **b**), the percentage of γH2AX positive nuclei are plotted (mean ± SD, ≥ 200 nuclei per test, *n* = 3 slides per condition, unpaired *t* test). **c**, **d** Scramble shRNA and EHMT1/2 double knockdown PEO1-OR cells were assayed for HR and NHEJ capacity using a two-plasmid GFP reporter assay. GFP+ cells percentage was determined by flow cytometry. **e**, **f** PEO1-OR cells were treated for 72 h with 1 μM UNC0638 or UNC0642 DMSO control, then assayed for HR and NHEJ capacity as in (**c**, **d**). GFP+ cell percentage is plotted (mean ± SD, 50,000 cells analyzed per test, *n* = 3, unpaired *t* test). **g** PEO1-OR cells were treated for 72 h with UNC0642, then the transcriptome was analyzed by RNA-Seq and compared to the transcriptome of four untreated PEO1-OR clones. Genes are plotted by fold change and *p* value. Red dots represent genes with false discovery rate (FDR) < 0.001 and absolute value of Log2 fold change > 0.5

EHMT1/2 have been implicated in direct roles in DNA repair by HR and NHEJ [18, 19]. We examined the functions of EHMT1/2 in promoting DNA repair in the context of PARPi-resistant HGSOC. We first examined whether EHMT1/2 disruption promoted increased DNA damage. We noted in PEO1-OR cells that EHMT1/2 knockdown resulted in a significant increase in DNA damage measured via immunofluorescent staining for phosphorylated histone H2AX (γH2AX) (Fig. 6a). Similar results were observed following 72-h treatment of PEO1-OR cells with EHMT1/2 inhibitors UNC0642 or UNC0638 (Fig. 6b) [27]. Similar treatment of PARPi-sensitive PEO1 parental cells showed higher baseline levels of γH2AX+ cells, but no difference between UNC0642-treated and untreated cells (Additional file 2: Figure S9A). Consistent with our dose response colony formation assays, these data suggest that PEO1-OR cells have a greater capacity to repair DNA damage than the sensitive parental PEO1, but that they may have a dependence on EHMT1/2 that is not present in the parental line. We next examined whether EHMT1/2 disruption in PEO1-OR reduces DNA repair using a two-plasmid system to evaluate NHEJ and HR repair pathways [28]. The readout of this assay is a functional GFP ORF, which we detected by flow cytometry. We observed a significant decrease in NHEJ and HR-mediated DNA repair in PEO1-OR shEHMT1/2 compared to shControl cells (Fig. 6c, d). Inhibition of EHMT1/2 with UNC0638 or UNC0642 also conveyed a significant reduction in both NHEJ and HR repair (Fig. 6e, f) in PEO1-OR cells. These data indicate that EHMT1/2 ablation is potentially conveying olaparib sensitivity through increased DNA damage. NHEJ was not affected by UNC0642 treatment in olaparib-sensitive PEO1 cells (Additional file 2: Figure S9B), further suggesting that the resistant PEO1-OR cells may have acquired a dependence on EHMT1/2. Parental PEO1 cells are known to be HR-deficient [23] and were therefore not tested for EHMT1/2-dependent effects on HR.

To identify genes and pathways regulated by EHMT1/2 in PARPi-resistant HGSOC cells, we treated PEO1-OR cells with UNC0642, then analyzed by RNA-Seq. We compared the transcriptomes of treated cells to four untreated clonal PEO1-OR populations and identified significantly changed gene expression (Fig. 6g). Consistent with the canonical function of EHMT1/2 and H3K9me2 as repressors of transcription, 78% of significantly changed genes were upregulated (2050 upregulated vs. 569 downregulated). We performed gene set enrichment analysis and identified overlap in numerous pathways. Consistent with our results, DNA repair pathways significantly overlapped with transcriptional changes. Notably, there was significant overlap with several pathways with unknown functions in the context of PARPi resistance, including PI3K, AKT, and mTOR. The top overlapping pathways are shown in Table 1, and their potential importance as resistance mechanisms is explored in the “Discussion” section.

**Table 1:** Gene set enrichment analysis of significantly changed gene expression in UNC0642-treated PEO1-OR HGSOC cells

Gene set name	# Genes in set (K)	Description	# Genes in overlap (k)	k/K %	*p* value	FDR *q* value
MTORC1 Signaling	200	Genes upregulated through activation of mTORC1 complex	43	22%	9.22E-20	4.61E-18
MYC Targets	200	A subgroup of genes regulated by MYC	41	21%	4.06E-18	1.02E-16
Unfolded Protein Response	113	Genes upregulated during unfolded protein response in the endoplasmic reticulum	30	27%	6.52E-17	1.09E-15
Hypoxia	200	Genes upregulated in response to hypoxia	39	20%	1.59E-16	1.98E-15
PI3K/AKT/ mTOR Signaling	105	Genes upregulated by activation of the PI3K/AKT/mTOR pathway	28	27%	6.17E-16	6.17E-15
Cholesterol Homeostasis	74	Genes involved in cholesterol homeostasis	23	31%	6.18E-15	5.15E-14
Oxidative Phosphor-ylation	200	Genes involved in oxidative phosphorylation	36	18%	3.04E-14	1.90E-13
Xenobiotic Metabolism	200	Genes involved in processing of drugs and other xenobiotics	36	18%	3.04E-14	1.90E-13
DNA Repair	150	Genes involved in DNA repair	30	20%	2.37E-13	1.32E-12
IL2/STAT5 Signaling	200	Genes upregulated by STAT5 in response to IL2 stimulation	34	17%	8.56E-13	3.89E-12

PEO1-OR cells were treated with 500 nM UNC0642 for 72 h. RNA was isolated and analyzed by RNA-Seq. The transcriptome of treated cells was compared to the transcriptomes of four untreated PEO1-OR clonal populations to identify significantly changed gene expression. Gene set enrichment analysis of significantly changed genes was performed. The top 10 overlapping pathways are shown.

## Discussion

Recent trials show that PARPi have significant clinical benefit to both newly diagnosed and recurrent HGSOC cases, regardless of BRCA status [3–5]. Nearly all HGSOC patients are now eligible to receive PARPi, and there is a critical need to identify, understand, and specifically target mechanisms of PARPi resistance. We have previously reported on Wnt signaling as a mechanism of PARPi-resistance in HGSOC [23]. Our RNA-Seq data of UNC0642-treated PEO1 did not show Wnt signaling as a top hit in gene set enrichment analysis. It is unknown if Wnt signaling and the mechanisms in this report are linked, regulate one another, or are entirely independent. Ovarian cancer, especially at later and metastatic stages is a heterogeneous disease, and it must be noted that more than one mechanism of resistance may exist within a patient, a population of cells, or even a single tumor. Therefore, it is of key importance to identify as many mechanisms as possible to develop the best possible treatment options. Going forward, combinatorial approaches will certainly be more effective than single agents.

We observed that there was a significant shift in the epigenetic landscape of *BRCA2*-mutant PARPi-resistant HGSOC cells, with a prominent enrichment of H3K9me2. Transcriptome analysis of PARPi-resistant cells narrowed the focus to two specific histone methyltransferases, EHMT1/2, which were significantly upregulated in both a cell line model and an in vivo PDX model of PARPi resistance. EHMT1/2 are methyltransferases whose canonical function is to catalyze H3K9me2, a modification associated with transcriptional repression [13–15]. Both genetic and pharmacologic approaches of disrupting EHMT1/2 activity reduced H3K9me2 levels and resensitized cells to olaparib, suggesting that these enzymes and the H3K9me2 epigenetic modification are playing a role in promoting or maintaining resistance. Single knockdown of EHMT1 or EHMT2 moderately restored olaparib sensitivity, but double knockdown of EHMT1/2 promoted a stronger resensitization phenotype. This is consistent with the known overlapping methyltransferase activities of EHMT1 and EHMT2 [15]. Mechanistically, in the context of PARPi-resistant HGSOC cells, EHMT1/2 disruption significantly reduced functional HR and NHEJ DNA repair pathways, and promoted DNA damage. Disruption only moderately altered cell cycle regulation and did not cause increased apoptosis or induce senescence. However, EHMT1/2 inhibition caused significant transcriptional changes in PARPi-resistant HGSOC cells, particularly in growth and survival pathways. Whether changes in these pathways are responsible for reduced mitotic markers remains to be determined.

Restoration of DNA repair capacity through secondary BRCA mutations have been described as mechanisms of PARPi resistance [6–9], and our findings strongly indicate that EHMT1/2 overexpression and/or hyperactivity also augment DNA repair in PARPi-resistant HGSOC. Our RNA-Seq analysis of UNC0642-treated PEO1-OR cells indicates that “DNA repair” genes were significantly changed by treatment. EHMT1/2 may therefore regulate DNA repair through epigenetic control of transcription. In addition to canonical roles in epigenetic regulation, several reports have demonstrated direct promotion of DNA damage repair by EHMT1/2, and subsequent knockdown increases sensitivity to chemotherapeutic agents [18, 19]. Our observation that disruption of EHMT1/2 promoted increased γH2AX and prevented repair are consistent with these results, and may be due to inhibition EHMT1/2 in recruiting repair factors to double-strand breaks. Further analysis of repair factor recruitment and repair kinetics will be required to ascertain the importance of these direct roles in PARPi-resistant HGSOC. In addition to roles in DNA repair, the complex of EHMT1/2 and ZNF644 have also been directly implicated in replication fork stability [29]. By disrupting EHMT1/2 in PARPi-resistant cells, we may be causing replication fork stalling or instability, thus slowing or preventing completion of DNA synthesis and subsequent entry into mitosis. DNA fiber or combing analyses may reveal if replication forks are stalled in the context of EHMT1/2 disruption.

In our models, EHMT1/2 disruption did not induce senescence or increase apoptotic response, but we did observe moderate differences in cell cycle when PARPi-resistant cells were co-treated with PARPi and EHMT1/2 inhibitor. Specifically, we noted a decrease in the percentage of cells in G2 and a reduction of inducers and markers of mitosis. It remains unknown if these changes are due to DNA damage or other effects. However, our gene set enrichment analysis of UNC0642-treated PEO1-OR showed an overlap with several pro-survival signaling pathways including mTOR, AKT, PI3K, and MYC. Rather than causing cell death, which we did not observe, disrupted EHMT1/2 interaction with these pathways may potentially sensitize resistant HGSOC cells by preventing growth and survival signaling. It is unknown if EHMT1/2 are acting only as epigenetic regulators of these pathways, or has direct interactions. However, EHMT1/2 often functions in a multi-subunit complex and so it will also be important to identify additional interacting factors. Notably, a recent study by Tu et al. showed that EHMT2 interacts with c-MYC to drive transcriptional repression and tumorigenesis in breast cancer, and that EHMT2 inhibition was a potent suppressor of MYC-dependent tumor growth [30]. c-MYC is often amplified in ovarian cancers and has previously been proposed as a therapeutic target in platinum-resistant cases [31]. Further study is required to determine if EHMT1/2 interacts with MYC, or other pro-survival signaling pathways, in the context of PARPi-resistant ovarian cancer.

Our mass spectrometry profiling of histone modifications revealed increased H3K9me2 in PARPi-resistant cells relative to sensitive cells. This may be due to a broad, but moderate, increase in H3K9me2 across the entire genome. However, given the significant changes in transcriptional programming observed in our RNA-Seq data, we surmise that it is more likely that H3K9me2 is highly enriched at multiple specific gene loci. Further investigation, including ChIP-Seq, is required to reveal the specific genomic loci enriched for H3K9me2 and EHMT1/2 in PARPi-resistant cells. Combined with our RNA-Seq analyses, these data will indicate epigenetically regulated genes that are potential effectors of PARPi resistance. Notably, BRCA1/2-wildtype OVCA433-OR cells did not upregulate EHMT1/2, but did have elevated H3K9me2, suggesting that the direct roles of EHMT1/2 in DNA repair may not be required for resistance in cells with functional BRCA. However, upregulation of H3K9me2 and subsequent transcriptional reprogramming may be a broader resistance mechanism.

Targeting EHMT1/2 to overcome PARPi resistance is a novel approach. The EHMT1/2 inhibitor UNC0642 has suitable pharmacodynamic properties for in vivo studies [25], which will allow for testing whether targeting EHMT1/2-dependent PARPi resistance is a viable therapeutic strategy in animal models. A recent report found that EGFR-tyrosine kinase inhibitor (erlotinib) resistant non-small cell lung cancer (NSCLC) has increased EHMT2 expression. Moreover, the authors found that combining erlotinib with UNC0642 significantly reduced NSCLC tumor burden in a PDX mouse model [32]. Looking beyond roles in chemoresistance and toward a more broad therapeutic strategy, EHMT1/2 inhibition may become a key target for immunotherapies. Liu et al. showed that EHMT2 inhibition synergized with DNA methyltransferase (DNMT) inhibition in A2780 and CAOV3 ovarian cancer cell lines to induce “viral mimicry,” a state in which expression of endogenous retroviruses and innate antiviral response genes are activated [33]. Studies in appropriate immunocompetent animal models will be essential to determine if such activity can induce the host immune system to kill ovarian cancer cells in vivo.

## Conclusions

Our reported findings further highlight the potential of EHMT1/2 inhibition to overcome targeted therapy resistance and prevent cancer progression. In conclusion, the epigenetic landscape is contributing to PARPi resistance through upregulation of EHMT1/2 and targeting EHMT1/2 is a potential approach to managing PARPi resistant HGSOC.

## Methods

### Cell culture, shRNA, and lentivirus

Cell lines were obtained from the Gynecologic Tumor and Fluid Bank (GTFB) at the University of Colorado, and were authenticated at the University of Arizona Genomics Core using short tandem repeat DNA profiling. Regular Mycoplasma testing was performed using MycoLookOut PCR (Sigma). HGSOC lines were cultured in RPMI 1640 supplemented with 10% fetal bovine serum (FBS) and 1% penicillin/streptomycin. 293FT lentiviral packaging cells were cultured in DMEM supplemented with 10% FBS and 1% penicillin/streptomycin. All cells were grown at 37 °C supplied with 5% CO_2_. shRNA in pLKO.1 lentiviral vector plasmids were purchased from the University of Colorado Functional Genomics Facility. Sequences and The RNAi Consortium numbers are listed in Additional file 4: Table S1. A scrambled non-targeting shRNA was used as control (Sigma-Aldrich #SHC016). Lentivirus was packaged as previously described [34] in 293FT using third-generation packaging plasmids (Virapower, Invitrogen) with polyethyleneimene (PEI) transfection in a 1:3 DNA:PEI ratio. Culture supernatant was harvested at 48–72 h post-transfection and processed through 0.45 μM filters. Viruses encoded a puromycin resistance gene. Transduced HGSOC cells were selected in 1 μg/mL puromycin. Functional DNA repair plasmids (described below) encode a puromycin resistance gene and thus preclude the use of pLKO.1 vectors, which also encode puromycin resistance. Knockdowns for these experiments were thus performed using pLKO.1-blast (Addgene #26655), which encodes a blasticidin resistance gene in place of puromycin. shControl, shEHMT1#1, and shEHMT2#1 were cloned into pLKO.1-blast between AgeI and EcoRI restriction sites. Virus was produced and cells were transduced and selected as described, except that selection occurred in 1 μg/mL blasticidin.

### Colony formation assay

Cell lines were seeded in 24-well plates and treated with increasing doses of olaparib. Media and olaparib were changed every three days for 12 days or until control wells were confluent, whichever occurred first. Colonies were washed twice with PBS, then incubated in fixative (10% methanol and 10% acetic acid in PBS). Fixed colonies were stained with 0.4% crystal violet in PBS. After imaging, crystal violet was dissolved in fixative and absorbance was measured at 570 nm using a Molecular Devices SpectraMax M2e plate reader.

### Histone modification profiling

Profiling of histone modifications in olaparib-sensitive and -resistant cells was performed by the Northwestern University Proteomics Core. Briefly, we provided frozen pellets of 5 × 10^6^ PEO1 and PEO1-OR cells. Histone extracts were trypsin digested and histone residues were assayed as previously reported [35, 36] by liquid chromatography coupled to mass spectrometry using a TSQ Quantiva Ultra Triple Quadrupole Mass Spectrometer.

### Tissue microarray

A previously constructed tissue microarray comprised of serous tumors from ovarian cancer patients treated at the University of Colorado was provided by the GTFB (COMIRB #17-7788). Slides were immunohistochemically stained for H3K9me2. Slides were blinded and staining was manually scored from 0 to 3, including half units. Scores < 2 were considered “Low” while scores ≥ 2 were considered “High.” A Kaplan-Meier survival curve was generated by correlating scores to overall patient survival. Only primary, chemonaïve tumors were used for generating the K-M curve.

### PDX mouse model of PARPi-resistant HGSOC

All animal experiments were performed in accordance with the Guide for the Care and Use of Laboratory Animals and were approved by the University of Colorado IACUC. Primary ovarian cancer sample GTFB1009 (BRCA1/2-wildtype) was provided by the University of Colorado GTFB. Six to 8-week-old NOD SCID gamma (NSG) mice (Jackson Labs) were given intraperitoneal injections of 5 million GTFB1009 ascites cells each. Following a 7-day incubation period, mice were given once daily intraperitoneal injections of 50 mg/kg olaparib or vehicle control (10% 2-hydroxypropyl-β-cyclodextrin, Sigma-Aldrich #C0926) for 21 days. After treatment, tumors were allowed to recur, and then mice were euthanized and ascites were collected for analysis.

### RNA-sequencing of olaparib-sensitive PEO1 and olaparib-resistant PEO1-OR

RNA was isolated from PEO1 olaparib-sensitive (*n* = 2) and four PEO1 olaparib-resistant clones using RNeasy columns with on-column DNase digest (Qiagen). RNA quality was confirmed using an Agilent Tapestation and all RNA used for library preparation had a RIN > 9. Libraries were created using Illumina TruSEQ stranded mRNA library prep (#RS-122-2102). Strand-specific pair-ended libraries were pooled and run on HiSeq4000 (Illumina). Library creation and sequencing were performed at the University of Colorado Genomics Core. HISAT [37] was used for alignment against GRCh37 version of the human genome. Samples were normalized using transcripts per kilobase million (TPM) measurement and gene expression using the GRCh37 gene annotation was calculated using home-made scripts. Analysis was performed by the Division of Translational Bioinformatics and Cancer Systems Biology at the University of Colorado School of Medicine. Data have been deposited to NCBI GSE117765.

### RNA-sequencing of UNC0642-treated olaparib-resistant PEO1-OR

PEO1-OR cells were treated for 72 h with 500 nM UNC0642. RNA was isolated from cells using the RNeasy Plus Mini Kit (Qiagen). RNA quality was confirmed using an Agilent Tapestation and all RNA used for library preparation had a RIN > 9. Library preparation and sequencing were performed by Novogene Co, Ltd. Using Illumina reagents and the HiSeq platform. Analysis was performed as above. Data have been deposited to NCBI GSE135864.

### Reverse-transcriptase quantitative PCR

RNA was isolated from cells using the RNeasy Plus Mini Kit (Qiagen). mRNA expression was determined using SYBR green Luna One Step reverse-transcriptase quantitative PCR (RT-qPCR) Kit (New England BioLabs) on a C1000 Touch (Bio-Rad) or QuantStudio 6 (Applied Biosystems) thermocycler. Expression was quantified by the ΔΔCt method using target-specific and control primers. β-2-microglobulin (*B2M*) and Glyceraldehyde 3-phosphate dehydrogenase (*GAPDH*) were used as internal controls. mRNA-specific primers were designed to span exon-exon junctions to avoid detection of genomic DNA. Primer sequences are shown in Additional file 4: Table S2.

### Ovarian cancer dataset analysis

Publicly available ovarian cancer databases (GSE9899, GSE13813, and GSE1926) were examined for correlations between disease recurrence and chemoresistance and EHMT2 expression (Oncomine, ThermoFisher).

### Inhibitors and antibodies

Olaparib (#S1060), UNC0638 (#S8071), and UNC0642 (#S7230) were obtained from SelleckChem. Full details of antibodies and usage for immunoblotting and immunofluorescence are given in Additional file 4: Table S3. Alexa Fluor 488-conjugated donkey anti-mouse secondary antibody (Invitrogen #A21202, 1:1000) was used for immunofluorescence detection of γH2AX and H3K9me2.

### Immunoblotting

For histone blots, extracts were made using the Histone Extraction Kit (Abcam #ab113476). For total protein, cells were lysed and briefly sonicated in RIPA buffer (150 mM NaCl, 1% TritonX-100, 0.5% sodium deoxycholate, 0.1% SDS, 50 mM Tris pH 8.0) supplemented with complete EDTA-free protease inhibitors (Roche #11873580001) and phosphatase inhibitors NaF and NaV. Protein was separated by SDS-PAGE and transferred to PVDF membrane using the TransBlot Turbo (BioRad). Membranes were blocked for 1 h at room temperature. Primary antibody incubation was performed overnight at 4 °C. Membranes were washed three times for 5 min each in TBST (50 mM Tris pH 7.5, 150 mM NaCl, 0.1% Tween-20), then secondary antibodies were applied for one hour at room temperature. Membranes were washed again three times for 5 min each in TBST. For fluorescent detection, bands were visualized using the LI-COR Odyssey Imaging System. For HRP detection, chemiluminescent signal was detected with SuperSignal West Femto (Thermo Scientific #34095) and visualized using a G:Box (SYNGENE). Details of blocking buffers and detection methods are provided in Additional file 4: Table S3.

### Densitometry

For immunoblot images captured using the LI-COR Odyssey, band fluorescence intensity was analyzed using LI-COR ImageStudio 4. For all others, G:Box images of immunoblots were analyzed using ImageJ. Immunoblots of histone extracts were normalized to band intensity of total H3. Immunoblots of total protein lysates were normalized to intensity of β-actin.

### Immunofluorescence

Cells were seeded on glass coverslips and treated as described in the figure legends. After treatment, cells were washed three times in PBS, then fixed in 4% paraformaldehyde for 10 min at room temperature, followed by three additional PBS washes. Cells were permeabilized for 3 min at room temperature using 0.2% Triton X-100. Primary and secondary antibodies were diluted in 3% BSA/PBS. Primary antibody was applied for 2 h at room temperature, followed by three washes in 1% Triton X-100/PBS and one wash in PBS. Secondary antibody was applied for 1 h at room temperature in the dark. Cells were washed three times with PBS, then mounted on glass slides using SlowFade Gold antifade reagent with DAPI (Invitrogen #S36938). Slides were imaged using an Olympus FV-1000 microscope (University of Colorado Advanced Light Microscopy Core).

### Two-plasmid functional DNA repair assay

Two-plasmid functional assays were performed to assess distal non-homologous end joining and homology directed repair. Cells were stably transfected with pimEJ5GFP (NHEJ) or pDRGFP (HR) by maintenance in 0.5 μg/mL puromycin and subsequently transfected with I-SceI restriction enzyme. After 72 h, transfected cells were collected and examined using a Beckman Coulter Gallios 561 flow cytometer (Flow Cytometry Shared Resource, University of Colorado) to quantify GFP positive cells. pimEJ5GFP (Addgene #44026) was a gift from Jeremy Stark. pDRGFP (Addgene #26475) and pCBASceI (Addgene #26477) were gifts from Maria Jasin. To control for I-SceI transfection efficiency, DNA was isolated from cells remaining after flow analysis and qPCR was performed using primers specific for transfected I-SceI DNA. Primers for Claudin 4 (CLDN4) gDNA were used as a DNA loading control. Primer sequences are listed in Additional file 4: Table S2.

### Annexin V/propidium iodide assay

Phosphatidylserine externalization was detected using an Annexin V/propidium iodide (PI) staining kit (Life Technologies) following the manufacturer’s instructions. Annexin V/PI positive cells were detected using a Beckman Coulter Gallios 561 Flow Cytometer.

### Β-galactosidase staining for senescence

Following drug treatment, PEO1-OR cells were incubated with 33 μM C_12_FDG (5-dodecanoylaminofluorescein d-β-d-galactopyranoside, Cayman 25583) for 1 h at 37 °C. Fluorescent cleaved substrate was detected using a Beckman Coulter Gallios 561 Flow Cytometer.

### Cell cycle staining

PEO1-OR cells were incubated with the indicated concentrations of olaparib and/or UNC0642 for 72 h, then collected by trypsinization and washed once with PBS. Cells were then resuspended and fixed in ice cold 70% ethanol for 1 h at − 20 °C. Cells were washed once with PBS, then resuspended in 200 μL PI staining solution [1× PBS, 50 μg/mL RNase A (Thermo Scientific #EN0531), 50 μg/mL propidium iodide (Thermo Scientific #P3566)] for 30 min at 37 °C, then analyzed by flow cytometry using a Beckman Coulter Gallios 561 Flow Cytometer.

### Software and statistical analysis

Flow cytometry analysis was performed using FlowJo 10. Statistical analysis and calculation of *P* value was performed using GraphPad Prism 7. Quantitative data are expressed as mean ± SD unless otherwise stated. Two-tailed *t* test was used for single comparisons. Analysis of variance (ANOVA) with Fisher’s least significant difference (LSD) was used in multiple comparisons. For all statistical analyses, the level of significance was set at 0.05.

## Supplementary information

**Additional file 1.** Full results of mass spectrometry histone profiling of PEO1 (Sens) and PEO1-OR (Res) cells. Spreadsheet includes average percent of each modification, standard deviation, and coefficient of variance (CV).

**Additional file 2: Figure S1.** Immunoblot of histone modifications in PEO1 and PEO1-OR cells. Histone extracts from PEO1 and PEO1-OR cells were resolved by SDS-PAGE followed by transfer to PVDF membrane and blotting for the indicated histone modifications. Densitometry analysis of band intensity was performed and normalized to total H3. **Figure S2.** Representative images of H3K9me2 staining within tumor and stromal regions of the TMA. T=tumor, S=stroma. **Figure S3.** Short-term exposure to olaparib does not induce EHMT1/2 mRNA expression in PARPi-sensitive PEO1 parental cells. PEO1 cells were treated with vehicle control, or with 600 nM olaparib for the times shown. RNA was isolated and mRNA expression of *EHMT1* and *EHMT2* were examined by RT-qPCR and normalized to *GAPDH* control. Data are shown as mean ± SD. N = 3. * *p* = 0.04. **Figure S4.** Schematic of PDX mouse model to generate olaparib-resistant ascites. Following collection, RNA and protein were isolated from control- and olaparib-treated ascites and were subsequently examined for EHMT1/2 mRNA and protein expression. **Figure S5.** Analyses of *EHMT1* and *ZNF644* in advanced and chemoresistant HGSOC. Analyses are of *EHMT1* and *ZNF644* correspond with analysis of *EHMT2* in main Fig. 3. (A) *EHMT1* mRNA expression in Borderline vs. HGSOC tumors and by grade (GSE9899), and relative copy number by stage (GSE13813). (B) Same as A, but for *ZNF644*. **Figure S6.** Single knockdown of EHMT1 or EHMT2 is partially effective at reducing H3K9me2 and sensitizing PARPi-resistant HGSOC. PEO1-OR cells were stably transduced with lentivirus encoding single shRNA against EHMT1 or EHMT2 or a scrambled shRNA control. EHMT1 and EHMT2 expression were examined by (A) RT-qPCR and (B) immunoblot. (C) Histone extracts from single knockdown or control cells were resolved by SDS-PAGE, transferred to PVDF, and immunoblotted for H3K9me2. Band intensity was quantified by densitometry and compared to total H3 control. Olaparib dose response colony formation assays were performed comparing control cells to (D) single EHMT1 knockdown or (E) single EHMT2 knockdown. IC50 values are shown. **Figure S7.** Effects of EHMT1/2 inhibitor on sensitization varies by cell line and drug treatment. (A) Cisplatin sensitivity of PARPi-resistant PEO1-OR cells co-treated with 1 μM UNC0642 was analyzed by dose response colony formation assays and compared to DMSO control treatment. Colony formation is plotted as mean ± SD of 3 wells. (B) Olaparib sensitivity of PARPi-sensitive PEO1 parental cells co-treated with 1 μM UNC0642 was analyzed by dose response colony formation assays and compared to DMSO control treatment. Colony formation is plotted as mean ± SD of 3 wells. (C) OVCA433 (*TP53*-mutant, *BRCA1/2*-wildtype) and OVCA433-OR cells were analyzed by Western blot for EHMT1, EHMT2, and Actin. (D) OVCA433 and OVCA433-OR cells were analyzed for H3K9me2 and H3. Densitometry analysis of the H3K9me2:H3 ratio is shown. (E) Olaparib sensitivity of PARPi-resistant OVCA433-OR cells co-treated with 1 μM UNC0642 was analyzed by dose response colony formation assays and compared to DMSO control treatment. Colony formation is plotted as mean ± SD of 3 wells. **Figure S8.** Densitometry analyses of cell cycle immunoblots following PARP and EHMT1/2 inhibition in PEO1-OR cells. Images of the immunoblots for the indicated proteins shown in main Fig. 5e were examined in ImageJ. Band intensity was quantified and normalized to Actin. Data are shown relative to Non-pretreated, control condition (white bar). **Figure S9.** DNA damage and repair are unaffected by UNC0642 in PARPi-sensitive PEO1 cells. (A) Correlates with main Fig. 6b. Immunofluorescence staining for DNA damage marker γH2AX was performed on PEO1 cells treated for 72 h with 1 μM UNC0642 or vehicle control. Nuclei were counterstained with DAPI. The percentage of γH2AX positive nuclei are plotted (mean ± SD, n=3 slides per condition, ≥200 cells per test, unpaired t-test). (B) Correlates with main Fig. 6e. PEO1 cells were treated for 72 h with 1 μM UNC0642 or vehicle control, then assayed for NHEJ capacity using a two-plasmid GFP reporter assay. GFP+ cell percentage was determined by flow cytometry and plotted (mean ± SD, 50,000 cells per test, n=3, unpaired t-test).

**Additional file 3:** Tumor Microarray Patient Characteristics (H3K9me2 staining and Kaplan-Meier analysis includes only 92 primary tumors prior to therapy).

**Additional file 4: Table S1.** shRNA. **Table S2.** Primers. **Table S3.** Antibodies.

## Data Availability

Data generated and/or analyzed during this study are available from the corresponding author on reasonable request. RNA-Seq data have been deposited to NCBI GSE117765 (PEO1 vs. PEO1-OR) and GSE135864 (PEO1-OR untreated vs. UNC0642 treatment). The Tothill (GSE9899), Etemadmoghadam (GSE13813), and Peters (GSE1926) cohort datasets are publicly available.

## Abbreviations

EHMT1/2: Euchromatic histone-lysine-*N*-methyltransferases 1 and 2
H3K9me2: Dimethylated histone H3 lysine 9
HGSOC: High-grade serous ovarian carcinoma
HR: Homologous recombination
NHEJ: Non-homologous end joining
PARP: Poly ADP ribose polymerase
PARPi: Poly ADP ribose polymerase inhibitor
PDX: Patient-derived xenograft
TMA: Tissue microarray
