# Supplementary material for: Histone methyltransferases EHMT1 and EHMT2 (GLP/G9A) maintain PARP inhibitor resistance in high-grade serous ovarian carcinoma
Source: Clin Epigenetics. 2019 Nov 27;11:165. doi: 10.1186/s13148-019-0758-2 (PMC6882350; doi:10.1186/s13148-019-0758-2)
Supplement: Supplementary file 2 — Additional file 2: Figure S1. Immunoblot of histone modifications in PEO1 and PEO1-OR cells. Histone extracts from PEO1 and PEO1-OR cells were resolved by SDS-PAGE followed by transfer to PVDF membrane and blotting for the indicated histone modifications. Densitometry analysis of band intensity was performed and normalized to total H3. Figure S2. Representative images of H3K9me2 staining within tumor and stromal regions of the TMA. T=tumor, S=stroma. Figure S3. Short-term exposure to olaparib does not induce EHMT1/2 mRNA expression in PARPi-sensitive PEO1 parental cells. PEO1 cells were treated with vehicle control, or with 600 nM olaparib for the times shown. RNA was isolated and mRNA expression of EHMT1 and EHMT2 were examined by RT-qPCR and normalized to GAPDH control. Data are shown as mean ± SD. N = 3. * p = 0.04. Figure S4. Schematic of PDX mouse model to generate olaparib-resistant ascites. Following collection, RNA and protein were isolated from control- and olaparib-treated ascites and were subsequently examined for EHMT1/2 mRNA and protein expression. Figure S5. Analyses of EHMT1 and ZNF644 in advanced and chemoresistant HGSOC. Analyses are of EHMT1 and ZNF644 correspond with analysis of EHMT2 in main Fig. 3. (A) EHMT1 mRNA expression in Borderline vs. HGSOC tumors and by grade (GSE9899), and relative copy number by stage (GSE13813). (B) Same as A, but for ZNF644. Figure S6. Single knockdown of EHMT1 or EHMT2 is partially effective at reducing H3K9me2 and sensitizing PARPi-resistant HGSOC. PEO1-OR cells were stably transduced with lentivirus encoding single shRNA against EHMT1 or EHMT2 or a scrambled shRNA control. EHMT1 and EHMT2 expression were examined by (A) RT-qPCR and (B) immunoblot. (C) Histone extracts from single knockdown or control cells were resolved by SDS-PAGE, transferred to PVDF, and immunoblotted for H3K9me2. Band intensity was quantified by densitometry and compared to total H3 control. Olaparib dose response colony formation assays were [file 13148_2019_758_MOESM2_ESM.pdf]

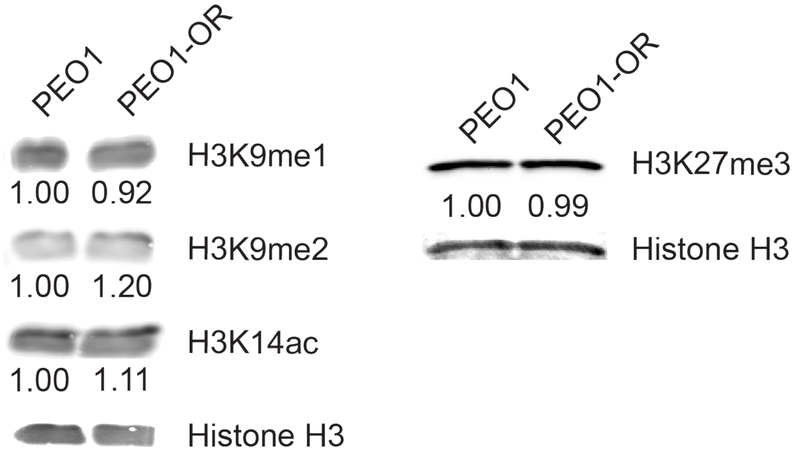

Fig. S1. Immunoblot of histone modifications in PEO1 and PEO1-OR cells. Histone extracts from PEO1 and PEO1-OR cells were resolved by SDS-PAGE followed by transfer to PVDF membrane and blotting for the indicated histone modifications. Densitometry analysis of band intensity was performed and normalized to total H3.

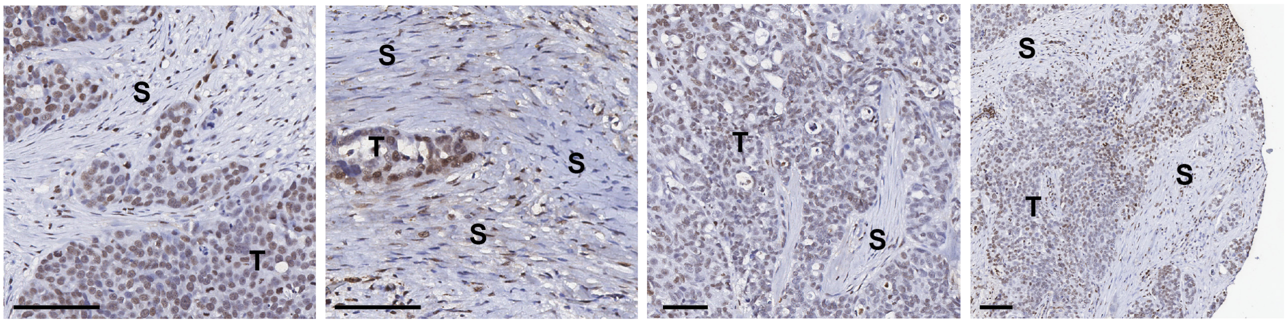

Fig. S2. Representative images of H3K9me2 staining within tumor and stromal regions of the TMA. T=tumor, S=stroma.

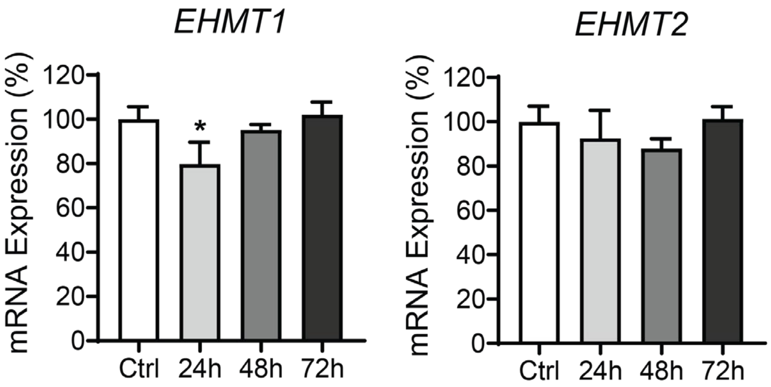

Fig. S3. Short-term exposure to olaparib does not induce EHMT1/2 mRNA expression in PARPi-sensitive PEO1 parental cells. PEO1 cells were treated with vehicle control, or with 600 nM olaparib for the times shown. RNA was isolated and mRNA expression of *EHMT1* and *EHMT2* were examined by RT-qPCR and normalized to *GAPDH* control. Data are shown as mean ± SD. N = 3. * p = 0.04.

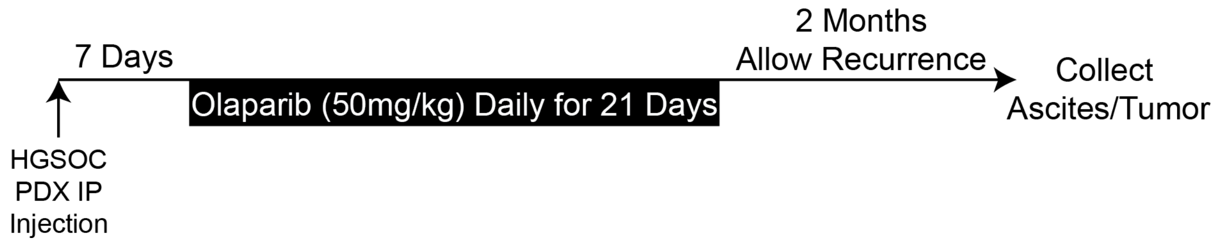

Fig. S4. Schematic of PDX mouse model to generate olaparib-resistant ascites. Following collection, RNA and protein were isolated from control- and olaparib-treated ascites and were subsequently examined for EHMT1/2 mRNA and protein expression.

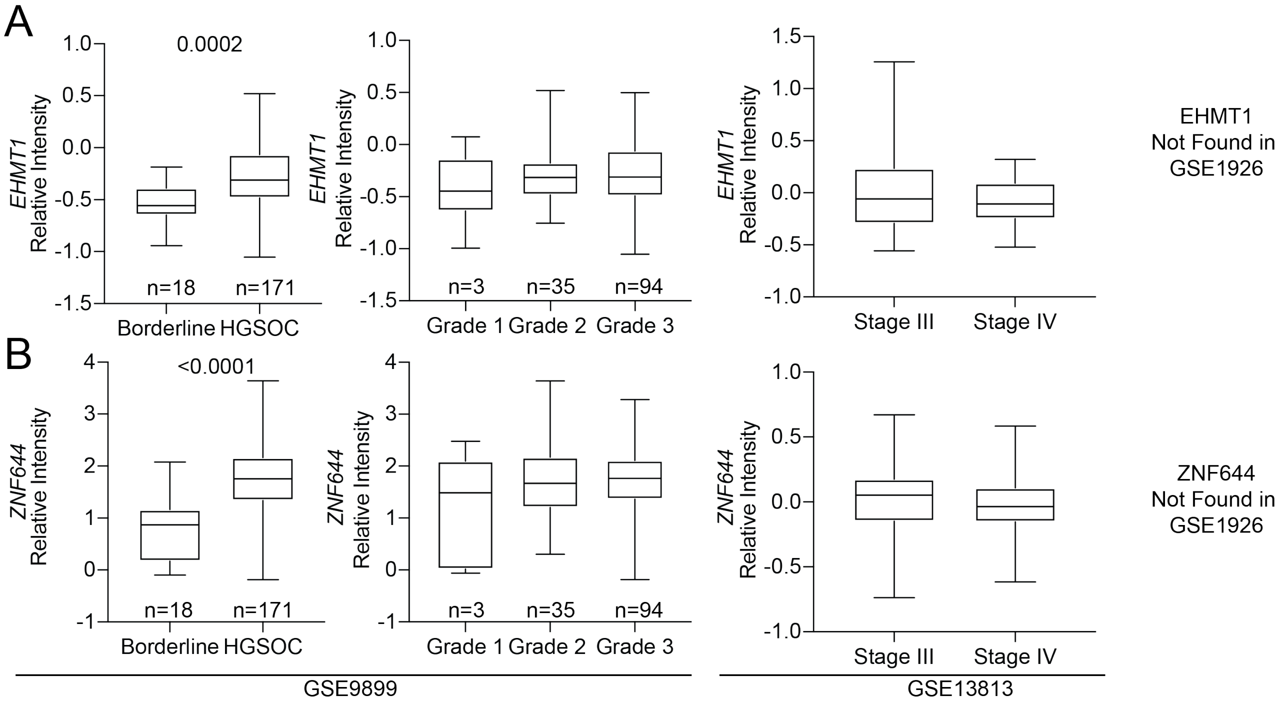

Fig. S5. Analyses of *EHMT1* and *ZNF644* in advanced and chemoresistant HGSOC. Analyses are of *EHMT1* and *ZNF644* correspond with analysis of *EHMT2* in main Fig. 3. **(A)** *EHMT1* mRNA expression in Borderline vs. HGSOC tumors and by grade (GSE9899), and relative copy number by stage (GSE13813). **(B)** Same as **A**, but for *ZNF644*.

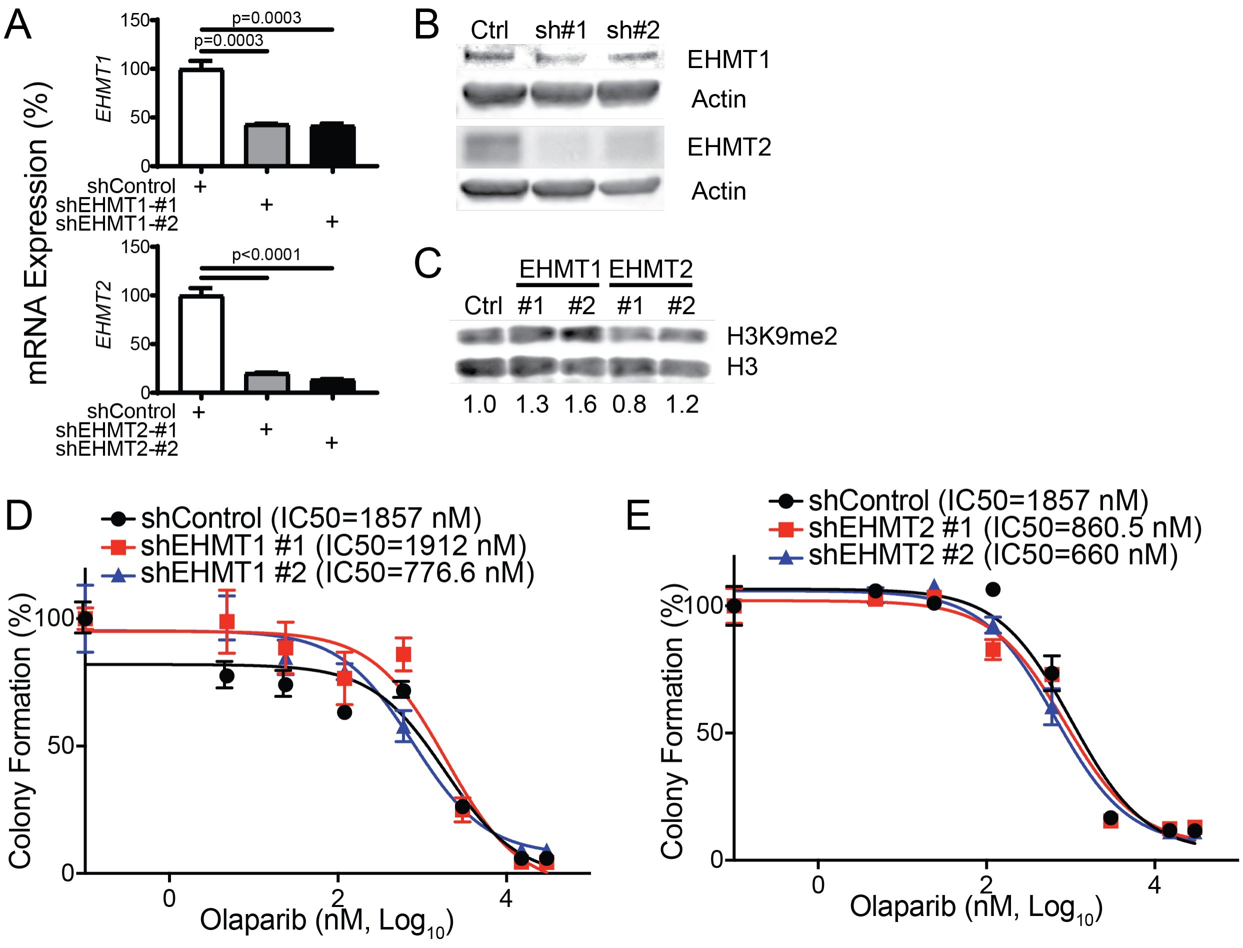

Fig. S6. Single knockdown of EHMT1 or EHMT2 is partially effective at reducing H3K9me2 and sensitizing PARPi-resistant HGSOC. PEO1-OR cells were stably transduced with lentivirus encoding single shRNA against EHMT1 or EHMT2 or a scrambled shRNA control. EHMT1 and EHMT2 expression were examined by **(A)** RT-qPCR and **(B)** immunoblot. **(C)** Histone extracts from single knockdown or control cells were resolved by SDS-PAGE, transferred to PVDF, and immunoblotted for H3K9me2. Band intensity was quantified by densitometry and compared to total H3 control. Olaparib dose response colony formation assays were performed comparing control cells to **(D)** single EHMT1 knockdown or **(E)** single EHMT2 knockdown. IC50 values are shown.

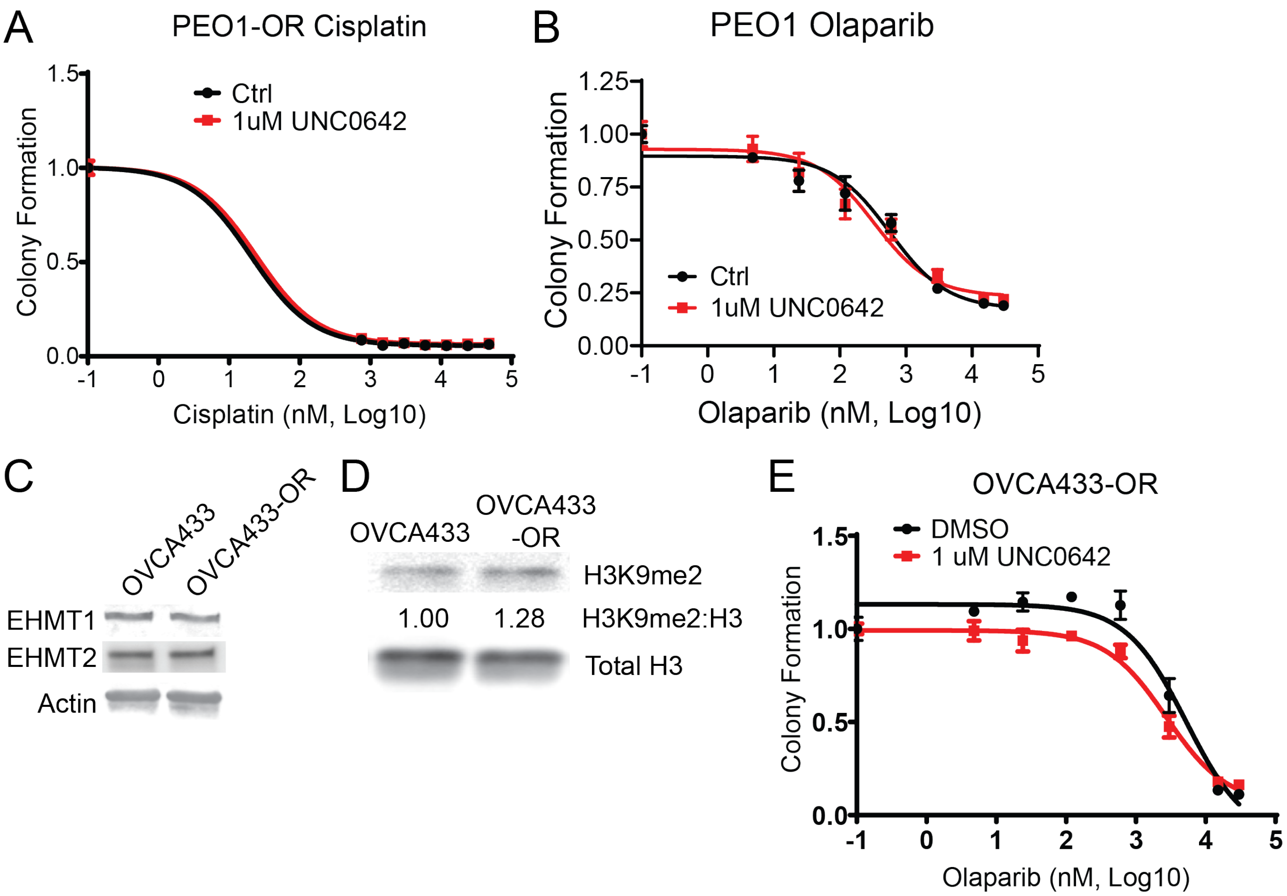

Fig. S7. Effects of EHMT1/2 inhibitor on sensitization varies by cell line and drug treatment. **(A)** Cisplatin sensitivity of PARPi-resistant PEO1-OR cells co-treated with 1 μM UNC0642 was analyzed by dose response colony formation assays and compared to DMSO control treatment. Colony formation is plotted as mean ± SD of 3 wells. **(B)** Olaparib sensitivity of PARPi-sensitive PEO1 parental cells co-treated with 1 μM UNC0642 was analyzed by dose response colony formation assays and compared to DMSO control treatment. Colony formation is plotted as mean ± SD of 3 wells. **(C)** OVCA433 (*TP53*-mutant, *BRCA1/2*-wildtype) and OVCA433-OR cells were analyzed by Western blot for EHMT1, EHMT2, and Actin. **(D)** OVCA433 and OVCA433-OR cells were analyzed for H3K9me2 and H3. Densitometry analysis of the H3K9me2:H3 ratio is shown. **(E)** Olaparib sensitivity of PARPi-resistant OVCA433-OR cells co-treated with 1 μM UNC0642 was analyzed by dose response colony formation assays and compared to DMSO control treatment. Colony formation is plotted as mean ± SD of 3 wells.

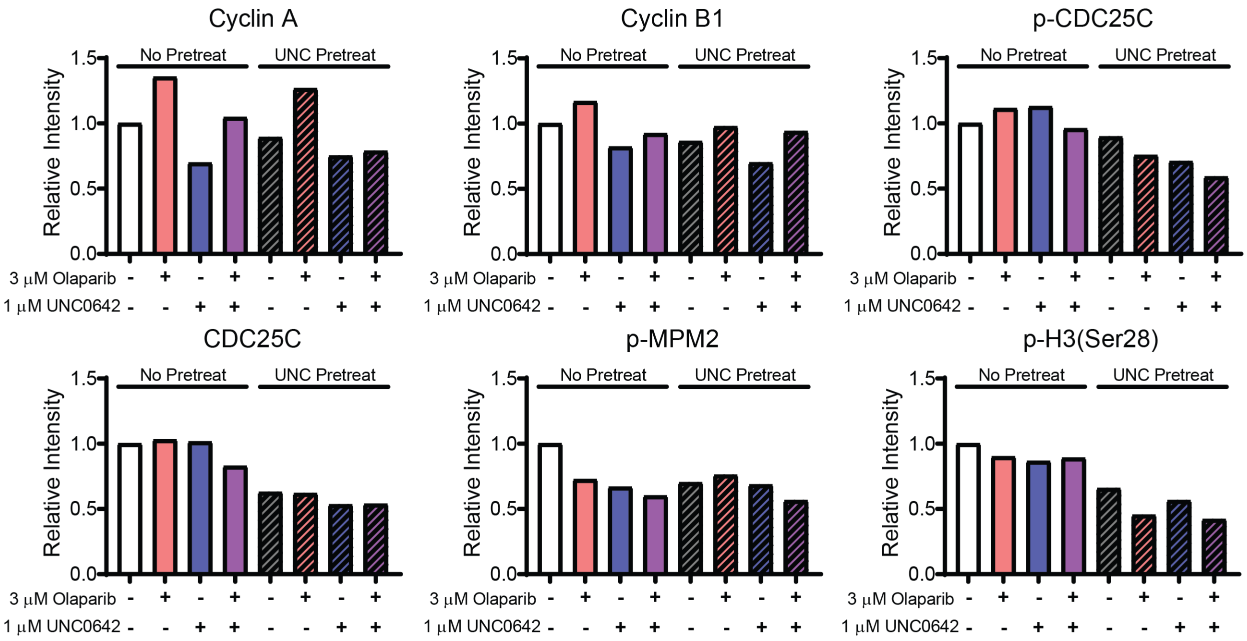

Fig. S8. Densitometry analyses of cell cycle immunoblots following PARP and EHMT1/2 inhibition in PEO1-OR cells. Images of the immunoblots for the indicated proteins shown in main Fig. 5E were examined in ImageJ. Band intensity was quantified and normalized to Actin. Data are shown relative to Non-pretreated, control condition (white bar).

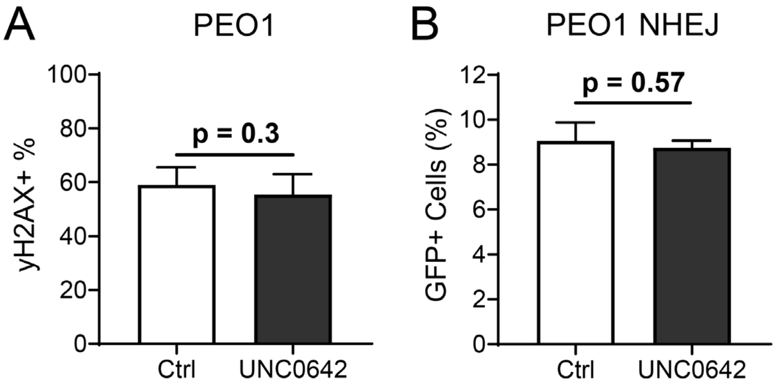

Figure S9. DNA damage and repair are unaffected by UNC0642 in PARPi-sensitive PEO1 cells. (A) Correlates with main Fig. 6B. Immunofluorescence staining for DNA damage marker γH2AX was performed on PEO1 cells treated for 72 h with 1 μM UNC0642 or vehicle control. Nuclei were counterstained with DAPI. The percentage of γH2AX positive nuclei are plotted (mean ± SD, n=3 slides per condition, ≥200 cells per test, unpaired t-test). **(B)** Correlates with main Fig. 6E. PEO1 cells were treated for 72 h with 1 μM UNC0642 or vehicle control, then assayed for NHEJ capacity using a two-plasmid GFP reporter assay. GFP+ cell percentage was determined by flow cytometry and plotted (mean ± SD, 50,000 cells per test, n=3, unpaired t-test).
