# Supplementary material for: Histone methyltransferases EHMT1 and EHMT2 (GLP/G9A) maintain PARP inhibitor resistance in high-grade serous ovarian carcinoma
Source: Clin Epigenetics. 2019 Nov 27;11:165. doi: 10.1186/s13148-019-0758-2 (PMC6882350; doi:10.1186/s13148-019-0758-2)
Supplement: Supplementary file 4 — Additional file 4: Table S1. shRNA. Table S2. Primers. Table S3. Antibodies. [file 13148_2019_758_MOESM4_ESM.pdf]

Supplementary Table 1. shRNA

shRNA	The RNAi Consortium No.	pLKO.1 Insert Sequence
shControl	N/A (Sigma-Aldrich #SHC016)	CCGGGCGCGATAGCGCTAATAATTTCTCGAGAAATTATTAGCGCTATCGCGCTTTTT
shEHMT1#1	TRCN0000229325	CCGGCCTCGGTTCTGAGTCGTATAACTCGAGTTATACGACTCAGAACCGAGGTTTTTG
shEHMT1#2	TRCN0000229326	CCGGGGATTCAGATGTCACCTTAAACTCGAGTTTAAGGTGACATCTGAATCCTTTTTG
shEHMT2#1	TRCN0000115667	CCGGCACACATTCCTGACCAGAGATCTCGAGATCTCTGGTCAGGAATGTGTGTTTTTG
shEHMT2#2	TRCN0000115670	CCGGCGAGAGAGTTCATGGCTCTTTCTCGAGAAAGAGCCATGAACTCTCTCGTTTTTG

Supplementary Table 2. Primers

Name	Sequence	Usage
B2M-F	GGCATTCCTGAAGCTGACA	Control (ΔΔCt)
B2M-R	CTTCAATGTCGGATGGATGAAAC	Control (ΔΔCt)
EHMT1-F	CCTCGACTCGGAAAAACCCA	Gene expression
EHMT1-R	AGTTGGGGTCAATTCCGTCC	Gene expression
EHMT2-F	ATAGCAAGGAGGAGGACGGTT	Gene expression
EHMT2-R	CCTCGATGTGCTTGTGCTCT	Gene expression
GAPDH-F	GTCTCCTCTGACTTCAACAGCG	Control (ΔΔCt)
GAPDH-R	ACCACCCTGTTGCTGTAGCCAA	Control (ΔΔCt)
ZNF644-F	ATTCTCCGATGCAGGTTTTGTG	Gene expression
ZNF644-R	GCTGCTTCGGCCATTAGTAGA	Gene expression
I-SceI-F	CCGTTCTCGTGATGAAGGTAAA	Transfection efficiency control
I-SceI-R	CACTGATCGTACAGCAGACATAC	Transfection efficiency control
CLDN4-F	GCCTTACTCCGCCAAGTATT	gDNA loading control
CLDN4-R	AGGGAAGAACAAAGCAGAGAG	gDNA loading control

Supplementary Table 3. Antibodies

Protein	Species	Supplier and Catalog #	Use	Dilution	WB Blocking	WB Detection
Total H3	Rb	Cell Signaling 4499	WB	1:1000	LI-COR[1]	Fluorescent[3]
H3K9me1	Rb	Millipore 07-450	WB	1:1000	LI-COR	Fluorescent
H3K9me2	Ms	Abcam ab1220	WB<br>IF	1:1000<br>1:200	LI-COR	Fluorescent
H3K14ac	Rb	Cell Signaling 7627	WB	1:1000	LI-COR	Fluorescent
H3K27me3	Rb	Cell Signaling 9733	WB	1:1000	LI-COR	Fluorescent
γH2AX	Ms	Millipore 05-636	IF	1:400		
B-actin	Ms	Abcam ab6276	WB	1:10,000	LI-COR	Fluorescent
EHMT1	Rb	Bethyl A301-642A	WB	1:500	5% BSA/TBST[2]	HRP[4]
EHMT2	Rb	Cell Signaling 3306	WB	1:1000	5% BSA/TBST	HRP
CDC25C	Rb	Cell Signaling 4688	WB	1:1000	5% BSA/TBST	HRP
p-CDC25C (Ser216)	Rb	Cell Signaling 9528	WB	1:1000	5% BSA/TBST	HRP
Cyclin A	Ms	Neomarkers MS-473-P0	WB	1:1000	5% BSA/TBST	HRP
Cyclin B1	Ms	Santa Cruz SC-245	WB	1:1000	5% BSA/TBST	HRP
p-MPM2	Ms	Upstate 05-368	WB	1:1000	5% BSA/TBST	HRP
p-H3(Ser28)	Rb	Cell Signaling 9713	WB	1:1000	5% BSA/TBST	HRP

WB = Western Blot

IF = Immunofluorescence

[1] All blocking and incubations in Odyssey buffer (LI-COR #927-50000)

[2] Blocking and primary antibody incubation in 5% BSA/TBST. Secondary incubation in 5% milk/TBST.

[3] Detection by LI-COR fluorophore-labeled secondary goat anti-rabbit (IRDye 680RD or IRDye 800CW, #925-68071 or #926-32211) or goat anti-mouse (IRDye 680RD or IRDye 800CW, #926-68070 or #925-32210) antibodies. Used at 1:20,000 dilution.

[4] Detection by Cell Signaling HRP-conjugated secondary goat anti-rabbit (Cell Signaling #7074) or goat anti-mouse (Cell Signaling #7076). Used at 1:5000 dilution.
